# Genetic Footprints of Seasonal Fluctuating Selection: A Comparison With Established Selection Forms

**DOI:** 10.1093/gbe/evag082

**Published:** 2026-04-02

**Authors:** Olivia L Johnson, Raymond Tobler, Joshua M Schmidt, Christian D Huber

**Affiliations:** Department of Biology, Pennsylvania State University, University Park, PA, USA; School of Biological Sciences, University of Adelaide, Adelaide, Australia; Evolution of Cultural Diversity Initiative, The Australian National University, Canberra, Australia; Department of Ophthalmology, College of Medicine and Public Health, Flinders University, Adelaide, Australia; Department of Biology, Pennsylvania State University, University Park, PA, USA

**Keywords:** fluctuating selection, genomic signatures, population genetic simulation, balancing selection, positive selection

## Abstract

Fluctuating selection is frequently studied in natural populations by observing allele frequency trajectories over evolutionarily brief timeframes or through theoretical analyses of frequency dynamics. However, little is known of its effect on linked neutral diversity. Here, we simulate seasonally fluctuating selection at a single locus and characterize its genomic footprint using diversity, site-frequency spectrum (SFS), and haplotype-based statistics. We find that adaptively fluctuating loci exhibit distinct signals that depend on when in the seasonal cycle the population was sampled and whether sampling occurs early or late relative to the onset of the cyclic selection pressure. Compared with other modes of selection acting on a single locus, adaptively fluctuating loci show genomic signatures that are not shared by hard and soft selective sweeps but overlap considerably with heterozygote advantage. Leveraging linear discriminant analysis, we identify a combination of summary statistics that most effectively distinguish fluctuating selection from heterozygote advantage. Our findings shed light on the characteristic genomic signatures of fluctuating selection and imply that in-depth analyses of the long-term dynamics of adaptively fluctuating loci is achievable from sequence data collected from one or two time points across a selection cycle.

Significance Empirical evidence for temporally fluctuating selection has grown steadily over the past few decades, though little is currently known about the genomic footprint left by this mode of selection. By simulating and analysing the impact of an adaptive fluctuating locus on linked neutral diversity we reveal how this mode of selection differs from other common forms across evolutionary timescales and show that this depends on the time of sampling within the seasonal cycle. Our findings provide an initial view of the nuanced genomic signatures of fluctuating selection and introduce statistical strategies for distinguishing it from other modes of selection in population genetic studies. Importantly, this research shows that identification of adaptive fluctuating loci only requires sequence data from one or two time points within a selective cycle, facilitating analysis across a wide variety of species.

## Introduction

It has long been known that natural selection can distort patterns in surrounding neutral genetic variation (Smith and Haigh 1974; Clarke 1979; Holderegger et al. 2006; Kern and Hahn 2018; Charlesworth and Jensen 2021) that can be used to identify genomic loci targeted by selection. Informative distortions of neutral genetic variation include changes to nucleotide diversity, haplotype frequencies, and the site frequency spectrum, and statistics have been developed to identify genomic regions displaying these specific patterns (Watterson 1975; Tajima 1983, 1989; Oleksyk et al. 2010; Garud and Rosenberg 2015; Bitarello et al. 2018). The effects of positive and classical balancing selection on these statistics are well-documented. Positive selection, from a de novo or very low-frequency allele, is characterized by a significant decrease in diversity surrounding the selected site (Smith and Haigh 1974; Braverman et al. 1995), an excess of rare variants (Braverman et al. 1995; Fay and Wu 2000), and an increase in haplotype homozygosity (Sabeti et al. 2002; Voight et al. 2006). In contrast, an increase in diversity and an excess of intermediate frequency variants are a hallmark of balancing selection (Charlesworth et al. 1997; Charlesworth 2006).

Fluctuating selection, defined by variation in the direction of selection over time, is a form of balancing selection that is receiving increasing attention in empirical and theoretical studies, with a number of studies looking at the genome-wide impact of such loci (Buffalo and Coop 2020; Bertram 2021; Rudman et al. 2022) but whose local genomic impact remains poorly explored (Huerta-Sanchez et al. 2008; Taylor 2013; Wittmann et al. 2023). Previous empirical studies of fluctuating selection had been limited to phenotype observations until advances in next-generation sequencing allowed the examination of allele frequency fluctuations across time (Bell 2010; Johnson et al. 2023). Genetic evidence for fluctuating selection has now been documented across a wide range of species, driven by both predictable environmental cycles (e.g. seasonal changes) and more stochastic processes such as host-pathogen dynamics and episodic climatic events (Ludwig et al. 2015; Garcia-Elfring et al. 2021; Kelly 2022; Pfenninger et al. 2022; Pfenninger and Foucault 2022; Lynch et al. 2024; and reviewed in Johnson et al. 2023). Evidence in *Drosophila melanogaster* is particularly advanced, with studies of natural and experimental populations finding tens to thousands of alleles exhibiting seasonal fluctuations in frequency between 4% and 20% (Bergland et al. 2014; Glaser-Schmitt et al. 2021; Machado et al. 2021; Behrman and Schmidt 2022; Rudman et al. 2022; Nunez et al. 2024). In addition to these empirical studies, a number of theoretical models have been developed to identify the mechanisms underlying fluctuating selection and the dynamics of individual and multiple loci (Haldane and Jayakar 1963; Takahata et al. 1975; Gillespie 1978, 1997; Cvijović et al. 2015; Wittmann et al. 2017; Bertram and Masel 2019; Park and Kim 2019; Kim 2023; reviewed in Johnson et al. 2023).

Previous work on fluctuating selection has largely aimed to add ecological realism and identify and explain the large number of fluctuating SNPs observed in *Drosophila* and other species (Bergland et al. 2014; Machado et al. 2021; Kelly 2022; Pfenninger et al. 2022; Pfenninger and Foucault 2022; Rudman et al. 2022; Bitter et al. 2024), with comparatively little attention being paid to the indirect effects of fluctuating selection on patterns of surrounding neutral genetic variation (but see Barton 2000; Huerta-Sanchez et al. 2008; Taylor 2013; Wittmann et al. 2023). As a form of balancing selection, fluctuating selection maintains genetic diversity directly at selected and linked sites; however, recent theoretical analyses suggest that fluctuating selection can trigger decreases in diversity at weakly linked and unlinked sites on a genome-wide level that overwhelm increases close to selected loci (Wittmann et al. 2023). This effect has been confirmed by a multiyear survey of *Daphnia pulex*, which showed that temporal variation in selection coefficients at hundreds of sites across the genome was responsible for reducing levels of genome-wide diversity (Lynch et al. 2024).

Previous work on the effect of fluctuating selection on the site frequency spectrum (SFS) has used diffusion approximations and simulations to explore changes in the SFS under random, autocorrelated environmental fluctuations (Huerta-Sanchez et al. 2008). Fluctuating selection was seen to skew the SFS away from rare and intermediate alleles toward high-frequency alleles compared with neutral expectations, though this pattern is less pronounced on the folded SFS whereby statistics leveraging folded spectra, like Tajima's D (Tajima 1989), remain largely unaffected. Additional insights include the low power of SFS-based statistics to discriminate fluctuating selection from positive selection when the temporal variance in the selection coefficient is small (Huerta-Sanchez et al. 2008) and increased divergence observed at selected sites compared with neutral polymorphism that is similar to the effects of positive selection (Huerta-Sanchez et al. 2008; Gossmann et al. 2014). While these findings have advanced our understanding of the influence of fluctuating selection on genetic variation and population genetic inference, the effect of nonrandom oscillations, such as those seen in *Drosophila,* remain largely unexplored. Furthermore, fluctuating selection remains uncharacterized in the context of haplotype-based statistics, which could offer additional insights into the genetic signatures characterizing this type of selection and aid the development of statistical methods for its detection in empirical data.

Here, we investigate the effect of fluctuating selection on closely linked neutral variation. We used tree sequence recording to conduct fast and robust simulations of a seasonally fluctuating beneficial allele originating from a single de novo mutation, characterizing its impact on diversity-, SFS-, and haplotype-based statistics. To delineate genetic signatures specific to adaptive fluctuating loci, we also performed simulations of positively selected loci, in the form of hard and soft selective sweeps, and classical balancing selection represented by heterozygote advantage and used linear discriminant analysis (LDA) to identify a combination of statistics that best discriminate fluctuating selection from these other selection modes. We find that fluctuating selection is readily differentiated from positive selection at the window containing the selected site for all tested selection statistics, and that leveraging information from multiple statistics allows for differentiation of fluctuating selection from heterozygote advantage. Importantly, the genetic signals created by seasonally fluctuating selection varied depending on its sampling time during the seasonal cycle, improving our capacity to differentiate it from other selection types that do not vary across seasons when data sampling covers multiple time points. Moreover, signatures of early and long-term fluctuating selection can be clearly distinguished, enabling inferences about the onset of selection.

## Results

We used the population genetic simulator *SLiM* (Haller and Messer 2023; v 4.0.1) to simulate a 5 Mb sequence for a diploid, randomly mating population with scaled constant population sizes (across both seasons), recombination rates, and mutation rates, that broadly reflect natural populations of *Drosophila melanogaster* living in temperate environments (Comeron et al. 2012; Schrider et al. 2013; Keightley et al. 2014). Seasonality was modeled as a binary environment (i.e. switching between hot and cold temperatures), with an equal number of generations (10; left unscaled, see methods) and downscaled constant population size (10,000; unscaled 1 million) per season, with adaptive fluctuating loci modeled according to Wittmann and colleagues (Wittmann et al. 2023). We simulated seasonal fluctuating selection as a single locus starting from a de novo summer-favored mutation introduced at the beginning of the summer season, with the opposite allele being favored in the winter (Table 1). Each seasonal allele was modeled with one of four scaled selection coefficients (*s*; 1, 0.5, 0.1, 0.01), spanning the range of estimates of unscaled *s* (0.01, 0.005, 0.001, 0.0001) from empirical data of fluctuating adaptive loci (Bergland et al. 2014; Machado et al. 2021; Rudman et al. 2022; Bitter et al. 2024), and one of two dominance coefficients (*h*; 0.5, 0.6). These dominance coefficients capture the effects of additive and nonadditive dominance. We also tested *h* = 0.9 but found the patterns of selection followed the same trend as those of *h* = 0.6 (Figs. S1 and S2). To enhance the maintenance of these adaptively fluctuating loci, both *s* and *h* parameters were kept constant across seasons and successive years. We examined fluctuating selection at two timepoints, (i) immediately after the seasonal locus reached its stable equilibrium frequency (early equilibrium) and (ii) after selection had been at equilibrium for approximately 96,000 generations (long-term). The use of these two sampling points provides the opportunity to determine how the signature of selection differs based on whether neutral variation in genomic regions surrounding the adaptive fluctuating locus is at equilibrium or not. The signals of fluctuating selection at a constant early timepoint (2,570 generations) can be observed in the supplementary material (Fig. S3). 50 replicates were conducted for each combination of parameters and selection type.

**Table 1:** Fitness equations from Wittmann et al. (2023) and used to simulate seasonally fluctuating selection

Season	*ω*_WW_	*ω*_SW_	*ω*_SS_
Winter	1 + *s_w_*	1 + *h_w_s_w_*	1
Summer	1	1 + *h_s_s_s_*	1 + *s_s_*

The fitness equation used to calculate an individual's fitness depends on the season they are in and the genotype at the seasonal locus.

### Fluctuating Allele Frequency Trajectories

**Fig. 1.**
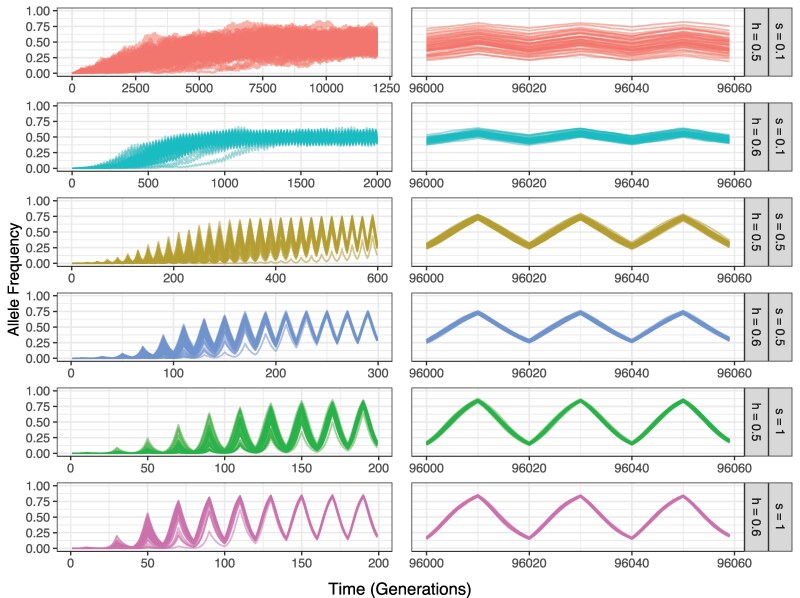
Allele frequency trajectories of fluctuating selection for different selection and dominance coefficients. Frequency of the summer-favored allele across time, shown for three different selection coefficients (*s*; 0.1, 0.5, 1) and two dominance coefficients (*h*; 0.5, 0.6) (labels on the right-hand side). Starting from a single mutation, the summer-favored allele gradually increases and both alleles oscillate around a mean frequency that eventually reaches a stable equilibrium, with alleles that have a higher dominance and stronger selection reaching their equilibrium value more quickly (left hand panels) and showing more consistent oscillatory behavior and lower stochastic allele frequency variance at equilibrium after 96,000 generations (right-hand panels). 50 replicates are depicted in each panel.

Our simulated adaptively fluctuating loci display a characteristic trajectory in which both alleles oscillate around an average frequency defined by the gradual increase of the summer allele toward a stable equilibrium (Fig. 1). For simulations with moderate to strong selection pressures (*s* ≥ 0.1) and nonadditive dominance (*h* > 0.5), the seasonal locus typically remained polymorphic in the population until the simulation terminated at 100,000 generations, though often became monomorphic before the end of the simulation under alternate parameterizations (i.e. *s* = 0.01 or *h* = 0.5). Accordingly, in subsequent analyses, we only consider simulations where the seasonal locus was segregating until the end of the simulation, with simulations restarted if the selected locus became monomorphic until this was achieved (Appendix 1). Among the resulting set of polymorphic seasonal loci, patterns followed expectations from studies by Wittmann et al. (2017, 2023), with stronger selection coefficients producing allele frequency fluctuations of larger amplitude and reduced stochastic variance due to drift, and loci with nonadditive dominance (*h* = 0.6) leading to more stable fluctuations than additive loci (*h* = 0.5; Fig. 1). When comparing these amplitudes of fluctuation to empirical datasets, we find that we capture a range of plausible scenarios with our simulated parameters. When *s* is 0.1, we observe a mean amplitude of fluctuation of 0.12; many empirical studies report average fluctuations around this range (Bergland et al. 2014; Machado et al. 2021; Rudman et al. 2022; Bitter et al. 2024). When *s* is 0.5, we see fluctuations in allele frequency of 0.46, which corresponds to the largest single allele frequency fluctuations observed in the datatsets of Bergland et al. (2014) and Machado et al. (2021) which were 0.37 and 0.45 respectively (Machado et al. 2021; Bergland et al. 2014). Finally, for *s* = 1 we observe allele-frequency fluctuations with much larger amplitude (0.68) than is usually reported empirically. While this regime is likely rare in natural populations, it is useful for visualizing the effects of strong seasonal selection on surrounding neutral diversity.

### Characterizing the Signatures of Fluctuating Selection

We characterized the impact of an adaptive seasonally fluctuating locus on linked neutral diversity across a 5 Mb simulated region by computing a suite of population genetic statistics across successive 10 kb windows (averaging over 50 replicates). Computed statistics include measures based on diversity, the site frequency spectrum (SFS), and haplotype homozygosity. We simulated loci under three scaled selection coefficients (*s* = 0.1, 0.5, 1) and a dominance coefficient of 0.6, as the latter resulted in more stable trajectories than purely additive loci. Unless otherwise specified, all reported results are based on samples taken from the final simulated summer generation where we expect the most distinction from neutrality due to the high stability of allele frequency at the selected site at this time point.

### Diversity Statistics

**Fig. 2.**
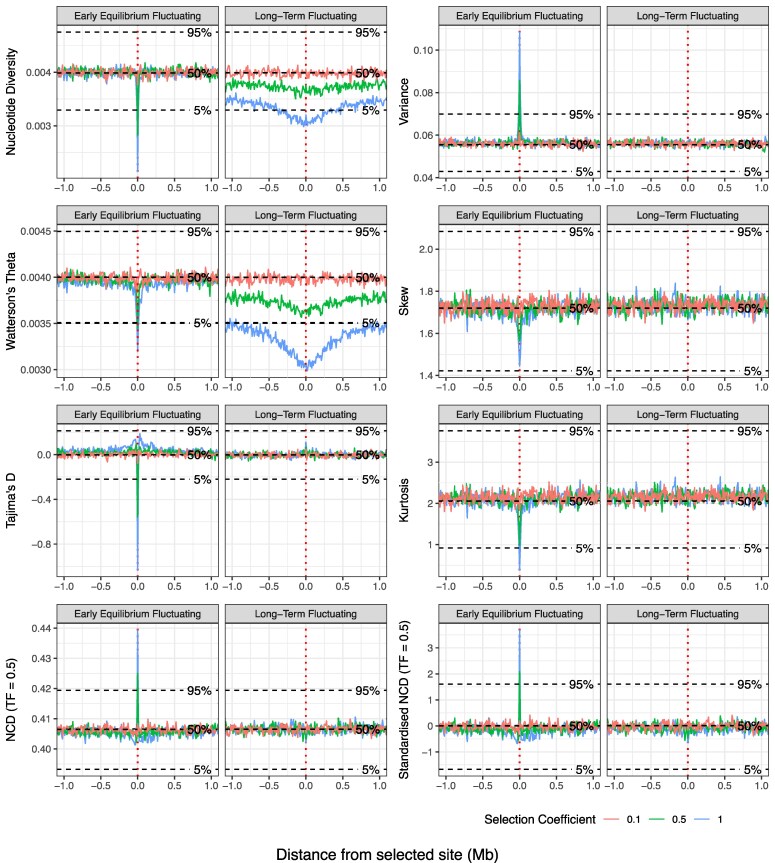
Footprints of fluctuating selection across time using SFS-based statistics. Signatures of fluctuating selection for a range of statistics shortly after stable frequency changes are achieved (early equilibrium) and several thousands of generations later when adaptive loci have been at equilibrium for an extended period of time (long-term). Statistics include nucleotide diversity, Watterson's theta, Tajima's D, noncentral deviation (NCD; basic and standardized by the number of segregating sites per window), as well as the variance, skew, and kurtosis of the SFS and are calculated from data sampled at the end of the summer season. The target frequency was set at 0.5 for basic and standardized forms of the NCD statistic. Statistics were calculated in 10 kb windows across the simulated region and were averaged across 50 replicates for three selection coefficients: 1; 0.5; and 0.1, (see key). The vertical dashed red line signifies the position of the selected site. Horizontal dashed lines illustrate the 5%, 50%, and 95% quantiles of the 50 replicate values of each statistic for neutral simulations (denoted by label).

When examining diversity measures including nucleotide diversity (π) and Watterson's theta (Fig. 2, Fig. S4), a narrow decrease in each statistic is observed around the adaptive fluctuating locus at the early equilibrium stage of selection, which becomes less pronounced with lower selection coefficients. When selection continues to the long-term equilibrium stage, however, the decrease broadens to encompass a region > 1 Mb either side of the selected site, with this decrease lessening with selection strength and becoming negligible when *s* = 0.1. For Tajima's D, sharp decreases are observed near to the selected site at early equilibrium, with positive shoulders appearing on either side; however, the genetic impact is reduced to a small and narrow positive peak once fluctuating selection has reached the long-term equilibrium stage (Fig. S4).

### 2nd to 4th Order Moments of SFS

We also examined the 2nd to 4th moments (variance, skew, and kurtosis, respectively) of the unfolded SFS to identify if fluctuating selection had any unique effects on the distribution of neutral allele frequencies surrounding a seasonally selected site (Fig. 2). At early equilibrium, a narrow peak is observed for allele frequency variance that increases with greater selection coefficients, indicating that fluctuating selection increases allele frequency variation around the selected site relative to neutrality. For both the skew and kurtosis of the SFS, we see a narrow negative peak nested within a broader, more subtle decrease that both become more pronounced as the selection coefficient increases (Fig. 2, Fig. S4). The decrease in skew and kurtosis around the selected site implies that the SFS becomes less skewed or bimodally distributed (e.g. fewer low-frequency variants and more high-frequency variants; Fig. 2, Fig. S5) compared with surrounding regions. Huerta-Sanchez and colleagues observed a similar effect on the SFS under randomly fluctuating selection (Huerta-Sanchez et al. 2008), showing that both randomly fluctuating selection as in Huerta-Sanchez et al., and cyclically fluctuating selection as in our study produced fewer singletons and low-frequency variants and more high-frequency variants than expected under neutral evolution, leading to negative values of Tajima's D (Fig. 2, Fig. S5). When examining impacts on the SFS across genomic regions further away from the selected site (≥250 kb), we observe an increase in high frequency variants compared with the neutral case, and an increase in intermediate variants compared with fluctuating selection at the central window, leading to positive Tajima's D values at this position (Fig. 2, Fig. S5). This pattern likely results from the recurrent sweep-like behavior of seasonally fluctuating selection (Coop and Ralph 2012). In contrast to the patterns observed during the early equilibrium stage, the moments of the SFS at the central window cannot be differentiated between fluctuating selection and neutral evolution once long-term equilibrium is reached (Fig. 2). While examination of the SFS reveals a decrease in singletons and low-frequency variants at this time point (Fig. S5), this is not sufficiently impactful to move moment estimates away from neutral expectations.

### Noncentral Deviation Statistic

The noncentral deviation (NCD) statistic is designed to identify genomic regions impacted by balancing selection by quantifying SFS deviations from an expected balanced equilibrium frequency (i.e. the target frequency, TF; Bitarello et al. 2018). Here, we use a version of NCD (NCD1) that does not require fixed differences to an outgroup, and apply three target frequencies (0.5, 0.4, 0.3). Following recommendations from the NCD developers, to control for potential confounding we also calculated a standardized measure that adjusts for the number of segregating sites in each window (Bitarello et al. 2018).

Fluctuating selection has a consistent impact on both basic and standardized NCD statistics when measured at all three target frequencies (Fig. 2, Figs. S4 and S6). When fluctuating selection has reached early equilibrium, the NCD statistic is characterized by a large increase at the selected locus relative to neutral simulations with a small decrease immediately flanking the peak for greater selection coefficients (*s*  *>* 0.1; Fig. 2). The height of this centralized positive peak decreases with the selection coefficient. This signature is consistent across the different target frequencies tested with only changes in the scale observed (Fig. S6). In all versions of the NCD statistic, this peak transforms to a small negative decrease at the selected site once long-term equilibrium is reached, a signature expected for a locus under long-term balancing selection (Charlesworth 2006; Bitarello et al. 2018). As the selection coefficient decreases, this signature becomes less apparent and is no longer visible when *s* = 0.1.

### Haplotype Statistics

**Fig. 3.**
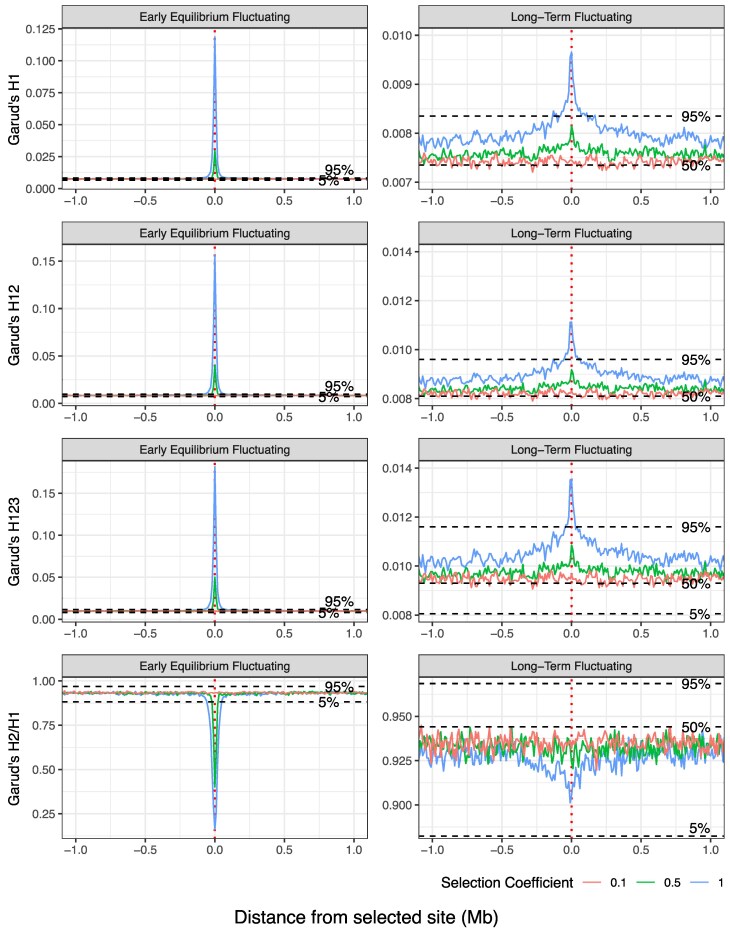
Signatures of fluctuating selection in haplotype statistics. Garud's H statistics calculated in 10 kb windows across a 2 Mb region centered upon the selected site for early equilibrium and long-term fluctuating selection. Samples were taken at the end of the summer season. Three selection coefficients were considered, *s* = 1, *s* = 0.5, and *s* = 0.1. Results show mean behavior at each 10 kb window, averaging over 50 replicates. Horizontal dashed lines illustrate the 5%, 50%, and 95% quantiles of the 50 replicate values of each statistic for neutral simulations (denoted by label). The 50% quantile is excluded from the Early Equilibrium panels due to the small range of the quantiles under these conditions.

Fluctuating selection also has a visible impact on haplotype-based statistics (Fig. 3). We calculated Garud's H statistics, which are a suite of statistics that measure distortions in the haplotype frequency distribution to identify regions under positive selection and discriminate between hard and soft sweeps. Computing Garud's H1, H12 and H123 on successive 10 kb windows, we observe a large narrow peak at the selected site for selection coefficients equal to 0.5 and 1 when fluctuating selection has reached early equilibrium. This peak becomes an order of magnitude smaller when selection has been stable and at long-term equilibrium. This distinctive positive peak in each statistic suggests that there is a small number of haplotypes at high frequencies surrounding the selected site, similar to a selective sweep. When looking directly at the frequency of the most common haplotypes, we see that the two most common haplotypes appear at an elevated frequency compared with the neutral case (Fig. S7). This signature is more pronounced for higher selection coefficients, which is also expected as stable oscillations are achieved more quickly as selection grows stronger, decreasing the time available for recombination and mutation to deteriorate the haplotype carrying the novel beneficial mutation. This results in a pattern that resembles a partial or incomplete hard selective sweep (noting that the winter-beneficial allele has little impact on the local haplotype frequency distribution despite being selected, as it was neutral prior to the selection period and is only weakly linked to neighboring neutral variants). Notably, Garud's H2/H1—i.e. the frequency of the second most common haplotype relative to the most frequent—shows a negative peak at the selected site at early equilibrium that becomes broader and less pronounced at the long-term equilibrium. In combination with the consistently high H12 values around the adaptive fluctuating locus, these patterns attest to an underlying haplotype frequency distribution indicative of a soft sweep (Garud and Rosenberg 2015) that becomes softer over time.

### Signatures of Fluctuating Selection Change Across the Seasonal Cycle

**Fig. 4.**
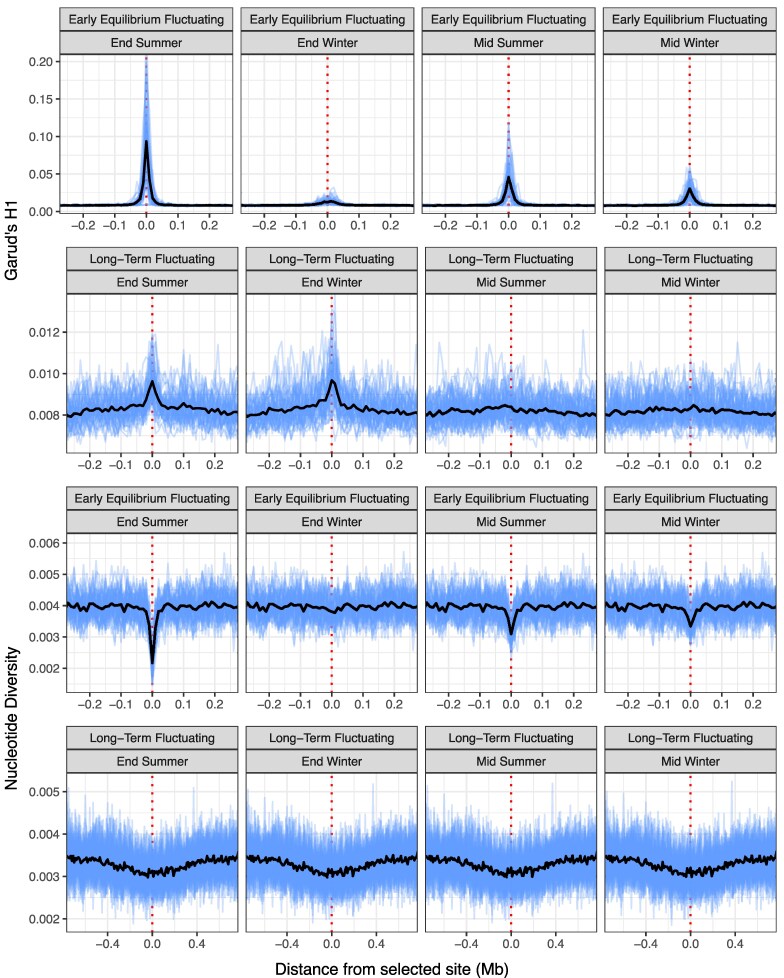
Signatures of fluctuating selection differ at different points of the seasonal cycle. Garud's H1 and nucleotide diversity values for early equilibrium and long-term fluctuating selection are plotted at the middle and end of summer and winter across a single seasonal cycle. Only data from simulated selection coefficients of *s* = 1 are shown as this produced the clearest patterns, with visualized statistics limited to Garud's H1 and nucleotide diversity as these reflect general patterns for SFS- and haplotype- based statistics, respectively (see Fig. S8).

While we have shown that fluctuating selection has a distinctive genomic footprint when sampled at the end of summer, the signature can change when sampling at different stages throughout the seasonal cycle. When sampling was performed at the end or midpoint of each season, distinct patterns appeared across all SFS and haplotype-based statistics (Fig. 4). These differences throughout the seasonal cycle are particularly pronounced at the early equilibrium stage, with signals being most distinct at the end of summer, before weakening in the middle of both seasons and becoming indistinguishable from neutral patterns by the end of winter (Fig. 4, Fig. S8). Notably, differences between seasons become negligible once long-term equilibrium has been achieved, though distinct features are present at the end of seasons that are not visible at seasonal midpoints for haplotype-based statistics (Fig. 4). In contrast, these within-season differences are not observed for diversity-based statistics (Fig. 4, Fig. S8).

Together, our results suggest that the optimal sampling time for detecting signatures of fluctuating selection in natural populations depends on a combination of the seasonal context of the newly arising adaptive allele, the duration of selection, and the statistic employed. In general, the highest statistical power is achieved by sampling closer to the end of the same season for which the new mutation is beneficial, with little to no detection power for these loci when samples are taken at the end of the opposite season. Sampling at seasonal midpoints tends to provide intermediate levels of power that depend more on the time of sampling and statistic employed, having limited value for haplotype-based statistics if loci have reached long-term equilibrium, but remaining useful for some SFS statistics throughout the post-equilibrium period (Fig. 4, Fig. S8).

### Distinguishing Fluctuating Selection From Other Forms of Selection

After characterizing the impacts of fluctuating selection on neighboring neutral variants across a range of SFS- and haplotype-based summary statistics, we compared these signals to those from more traditional forms of selection, such as hard and soft sweeps and classical balancing selection under heterozygote advantage. We aimed to ascertain if fluctuating selection has unique features that can be leveraged to discriminate it from other types of selection in empirical data. In keeping with our modeling of fluctuating selection, our simulations of heterozygote advantage and hard sweeps start from a single de novo mutation (Fig. S9), with heterozygote advantage being modeled as a symmetrically overdominant locus (assuming an equilibrium allele frequency of 0.5, corresponding to equal fitness for both homozygous genotypes). We also simulated soft selective sweeps, using a single locus model where numerous beneficial mutations contributing to the adaptive trait occur at the same site. This was implemented by increasing the mutation rate at the selected site, thereby introducing multiple beneficial mutations during each simulation. All forms of selection were simulated with a selection coefficient, *s*, of 0.1, with fluctuating and positively selected loci further simulated with *s* = 0.5 and 1. Heterozygote advantage was not simulated at the two highest strengths due to its unrealistic impacts on fitness which lead to large proportions of (heterozygous) individuals not contributing any offspring each generation. Dominance coefficients were set to 0.6 for fluctuating selection as this leads to stable trajectories (Fig. 1), and 0.5 for positive selection (i.e. additive allelic effects).

For hard sweeps, data was sampled immediately after fixation of the selected allele. For soft sweeps, data was sampled immediately after fixation of the beneficial trait, i.e. which occurs in the current context when at least two beneficial alleles from different mutational origins appear in each individual in the population. Heterozygote advantage (HA) and fluctuating selection (FS) were sampled after the adaptive loci either reached their equilibrium frequency (Early Equilibrium HA/FS; denoted by stable oscillations in the latter) and in the final sampling year of the simulation (Long-Term HA/FS; taken at ∼96,000 generations). At each timepoint we sampled individuals for 20 consecutive generations such that our samples covered a full seasonal cycle.

Population genetic statistics were again calculated in successive 10 kb windows across the simulated sequence. For both post-equilibrium sampling points, we used multiple-testing corrected *t*-tests to contrast signatures of the different forms of selection at the focal window, which is centered over the selected site, and calculated Cohen's D (Cohen 1988; Grice and Barrett 2014) to further evaluate the degree of overlap in the distribution of statistics. For all analyses, we used samples taken at the end of summer where we expect the strongest distinction between different selection models.

### Signals of Positive Selection are Distinct From Those of Fluctuating Selection

At the weakest simulated selection strength (*s* = 0.1), both hard and soft sweep patterns were significantly different (*P* < 0.05; Fig. S10) from fluctuating selection at both post-equilibrium sampling points for all statistics. The differentiation with hard selective sweeps was also apparent in measurements of Cohen's D, with no more than 5% overlap between distributions of nucleotide diversity and Watterson's theta (all *t*-test *P* < 0.001) and approximately 10% overlap observed for Tajima's D. Both basic and standardized NCD at all target frequencies displayed between 7% and 17% overlap (*P* < 0.001) and no more than 30% overlap was observed for the moments of the SFS (*P* < 0.001; Figs. S10 and S13; Grice and Barrett 2014). Considerably more overlap was observed for adaptively fluctuating loci and soft sweeps for these statistics, with Tajima's D overlapping by as much as 75%, all cases of NCD demonstrating between 60% and 70% of overlap, and diversity- and SFS-based measures ranging between 7% and 30% overlap. Distributional overlaps were less exaggerated for haplotype-based statistics (i.e. 10% to 40% overlap with hard sweeps and 40% to 60% overlap with soft sweeps).

These results suggest that at both post-equilibrium timepoints fluctuating selection tends to produce genetic signals that are closer to those generated by soft sweeps than hard sweeps (Fig. S10), though there was an exception to this general pattern. The skew and kurtosis of the SFS exhibit similar levels of overlap with both forms of positive selection (*P* < 0.001), overlapping 18% to 30% with hard sweeps and 24% to 27% with soft sweeps, as a result of these higher SFS moments being highly similar for hard and soft selective sweeps.

**Fig. 5.**
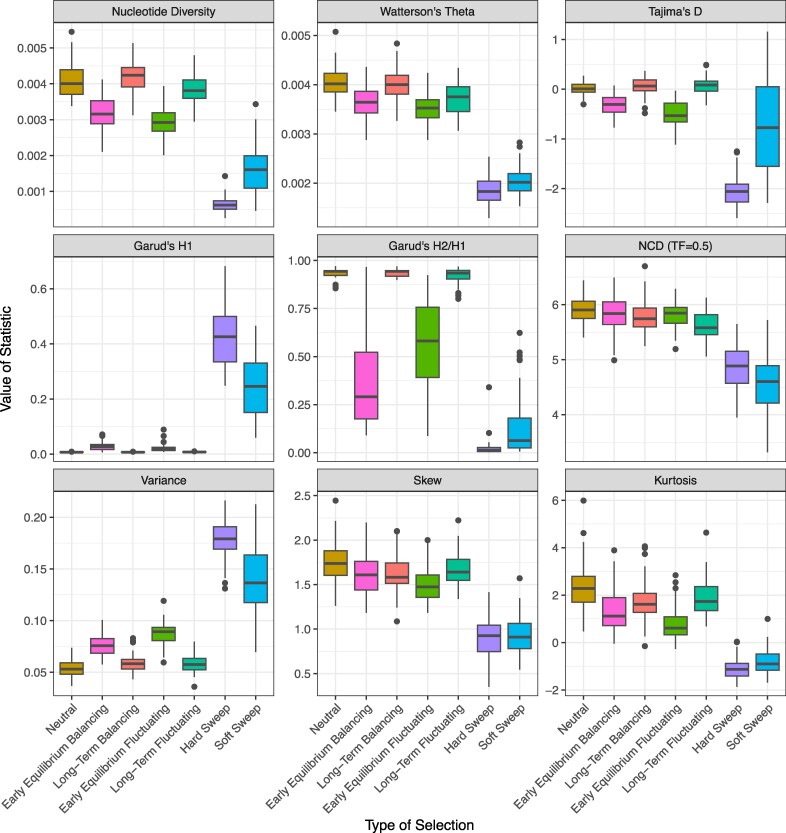
Population genetic statistics at the 10 kb window centered over the selected site for different modes of selection. Boxplots showing the distribution of values for different statistics, labeled at the top of each facet, evaluated for four modes of selection (see *x*-axis labels; 50 replicates and *s* = 0.5 for each selection coefficient excluding heterozygote advantage where *s* = 0.1). Positive selection in the form of hard and soft selective sweeps often cluster together and are distinct from Early Equilibrium and Long-Term Heterozygote Advantage (HA) and Fluctuating Selection (FS) and neutral evolution for most statistics.

These trends in the overlap between fluctuating and positive selection persist with stronger selection (*s* = 0.5 and *s* = 1; see Fig. 5, Figs. S11, S12, S14, and S15). In particular, differences between early equilibrium fluctuating selection and soft sweeps for *s* > 0.1 are insignificant for all comparisons of NCD (*P* > 0.05) but become distinct when fluctuating loci reach long-term equilibrium (*P* < 0.05). A similar behavior is observed for Tajima's D, with distinction between soft sweeps and strongly selected fluctuating loci only observed at long-term timepoints (*s* ≤ 0.5; *P* > 0.05).

Our results suggest that SFS patterns produced by soft selective sweeps are highly similar to those generated by fluctuating selection shortly after these loci reach equilibrium (i.e. positive standardized NCD, negative Tajima's D), whereas the signals of long-term fluctuating selection shift closer to genetic patterns expected of long-term balancing selection (negative standardized NCD, positive Tajima's D). More generally, nearly all statistics show significant distributional differences between fluctuating and positive selection however, weak positive selection produces distributions similar to strongly selected fluctuating loci for a handful of statistics (Fig. S16). For example, nucleotide diversity and the variance and kurtosis of the SFS are not significantly different between soft positive sweeps and fluctuating selection at early equilibrium when the selection strengths of the former (0.1) is an order of magnitude smaller than the latter (1), and comparisons of the Tajima's D and NCD are also nonsignificant between fluctuating selection and hard sweeps measured at these same time points and selection coefficients.

Importantly, while fluctuating selection is distinct from positive selection at both post-equilibrium timepoints for many statistics, it frequently did not differ from neutral evolution in our analysis, particularly when selection is relatively weak. Under the weakest tested selection scenario, *s* = 0.1, fluctuating selection at early equilibrium phase only significantly differed from neutral simulations for nucleotide diversity, Watterson's theta, and variance in the SFS (*P* < 0.01; Fig. S10), while only Tajima's D differed from neutral evolution at the long-term equilibrium stage (*P* < 0.05; Fig. S10). This suggests that while distinguishing fluctuating selection from positive selection is tenable for a variety of SFS and haplotype statistics, differentiating it from neutrality using the same set of statistics is more challenging with only SFS statistics able to make this distinction.

### Fluctuating Selection Shows Distinct but Subtle Differences From Heterozygote Advantage

To compare the impacts of fluctuating selection and heterozygote advantage on neighboring neutral variation, we simulated each using models outlined above (i.e. three selection coefficients for the former [0.1, 0.5, 1] and one [0.1] for the latter) and contrasted statistical distributions at their equivalent post-equilibrium time points sampled at the end of summer where the most differentiation is expected to occur.

At early equilibrium when *s* = 0.1, only SFS skew did not produce significant differences between heterozygote advantage and fluctuating selection (*P* > 0.05; Fig. S10), with Garud's haplotype statistics showing the maximum differentiation between the two selection forms (overlap between 13% and 35%; Fig. S17). Among the remaining significant statistics, the SFS variance also shows the next lowest levels of overlap (∼45%) than all other statistics which overlap across 61% to 80% of their distributions. Interestingly, the majority of statistics also show significant differences between fluctuating selection and heterozygote advantage at early equilibrium when *s* = 1 (*P* < 0.05; Fig. S12), though fewer occur when *s* = 0.5 (nucleotide diversity, Watterson's theta, variance of the SFS, basic and standardized NCD at all TF, and Tajima's D have *P*  *<* 0.05; Fig. S11). These patterns may arise because equilibrium is reached in a similar time frame for both heterozygote advantage (∼140 generations) and fluctuating selection (∼160 generations) when *s* = 0.5, resulting in similar genetic patterns at this sampling timepoint, which become increasingly different as the time to equilibrium also diverges.

In contrast to the patterns at early equilibrium, only nucleotide diversity and basic and standardized NCD (TF = 0.4 and 0.5) demonstrated a significant difference between the two forms at the long-term sampling point when *s* = 0.1 (*P* < 0.05), with haplotype-based and diversity statistics being significant at this time point when *s* = 0.5 and 1 (all *P* < 0.05; Fig. S11), while NCD becomes insignificant (*P* > 0.05). Together, this indicates that the two modes of balancing selection converge on similar, neutral SFS and haplotype distributions over ∼97,000 generations but the haplotype convergence might take longer for stronger selection strengths, in contrast the SFS converges quicker with greater selection coefficients (Fig. S18).

**Fig. 6.**
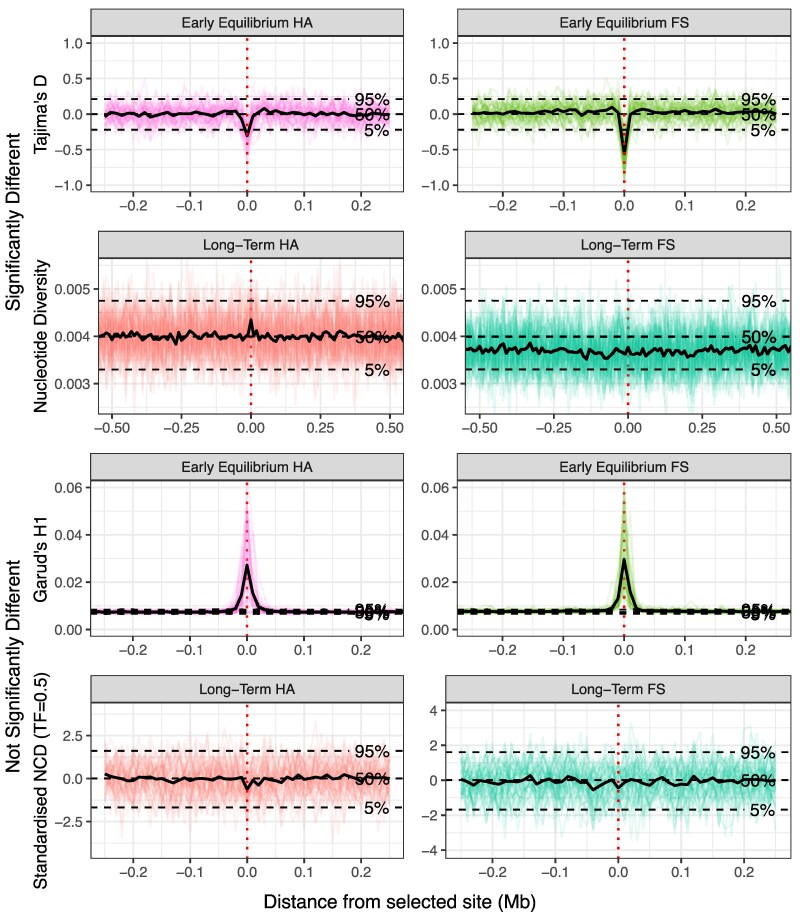
Comparisons between balancing and fluctuating selection. The values of the statistics for comparisons of fluctuating selection (*s* = 0.5) and heterozygote advantage (*s* = 0.1). The colored lines are the values of the replicates, while the black line shows the average value at each 10 kb window. Both early equilibrium and long-term sampling points are shown. The top two rows show statistics that are significantly different at the central window, while the bottom rows show statistics that demonstrate are not significantly different. The dashed vertical line shows the position of the selected site. Horizontal dashed lines illustrate the 5%, 50%, and 95% quantiles of the 50 replicate values of each statistic for neutral simulations (denoted by label).

If we consider the broader simulated region and when selection has similar outcomes for both heterozygote advantage (*s* = 0.1) and fluctuating selection (*s* = 0.5), signals consistent with a partial sweep are observed for both modes of balancing selection at early equilibrium (Fig. 6). This is expected, as in both cases selection acts upon a beneficial de novo mutation that is sampled immediately after the allele frequency becomes stable (i.e. averaging 140 generations for heterozygote advantage and 160 generations for fluctuating selection). More surprisingly, partial sweep signals did not tend to become more evident for fluctuating selection even though the beneficial allele reached greater frequencies than under heterozygote advantage. Both balancing selection modes tended to produce similar statistics at early equilibrium (and fluctuating loci actually produce less prominent peaks in Garud's H1 at this time point; Fig. 6, Fig. S19). The initial difference in time to reach equilibrium allele frequency, combined with the fluctuating allele trajectory, is likely causing these observations. Because heterozygote advantage reaches the equilibrium more quickly, there is less opportunity for recombination to act before equilibrium is established, compared with fluctuating selection at the same time point, resulting in a comparatively greater loss of diversity around the selected site for the former selection mode that is maintained until later in the simulation when recombination can break up the haplotype the selected allele arose upon. In addition, heterozygote advantage maintains this signal for longer than fluctuating selection, where it deteriorates faster due to the constant change in the direction of the allele frequency. This partial sweep signal weakens as the selection coefficient decreases and the alleles take more time to reach a balanced equilibrium (Fig. S20).

### Linear Discriminant Analysis

Our results show that the suite of summary statistics examined in this study capture changes in the distribution of neutral alleles around selected sites that have differential capacities to distinguish fluctuating selection from neutral evolution and other modes of selection at different evolutionary time points. While hard and soft sweeps are differentiable from fluctuating selection at the level of individual statistics, we conducted Linear Discriminant Analysis (LDA) using the “lda” function of the *r* package *MASS* to determine if some combination of the tested summary statistics could be used to discriminate fluctuating selection from neutral evolution and heterozygote advantage.

To perform this analysis, we created a training set by simulating 150 additional replicates for neutrally evolving sequences and adaptive loci under both fluctuating selection (for *s* = 0.1, 0.5, and 1) and heterozygote advantage (*s* = 0.1) using the original 50 simulations as validation or holdout datasets. Separate LDAs were conducted for fluctuating selection sampled at different seasonal timepoints, with data taken from the central 10 kb window and also from multiple windows in flanking regions to improve our discrimination ability. Each LDA compared data from neutrally evolving sequences and early and long-term equilibrium for both selection modes, with specific tests set up to explore the following four scenarios;

1. Fluctuating selection samples taken at the end of summer, with only the central window (i.e. containing the beneficial variant for selection simulations) being considered.
2. Fluctuating selection samples taken at the end of summer, with the central window and an immediately adjacent 10 kb window, plus a window 250 kb away from the selected site, being included.
3. Fluctuating selection samples taken at the end of both seasons, with only the central window being considered.
4. Fluctuating selection samples taken at the middle and end of summer, with only the central window being considered.

As there are a large number of statistics to be considered in each LDA, and not all may significantly contribute to discriminating the different forms of selection, we used a stepwise approach to first determine a set of statistics that allow for the best discrimination of selection types in the training dataset. The approach utilized the “greedy.wilks” function (of the *klaR* package) to determine which statistics contribute significantly to discriminating the different selection types and sampling points (i.e. early equilibrium vs. long-term). This function utilizes Wilks' lambda, a measure of how well groups can be separated based on dependent variables, and aims to minimize this value to determine which statistics will provide the best discriminatory ability (Wilks 1932). We first conducted stepwise forward variable selection on the training dataset of 150 replicates for each selection type and post-equilibrium time point. This returned a set of variables to be used in the subsequent LDA (Table S1). The resulting model was then used to predict both the mode of selection and the post-equilibrium time point of data sampling, with the remaining 50 replicates used to estimate the accuracy of the model through cross-validation.

**Fig. 7.**
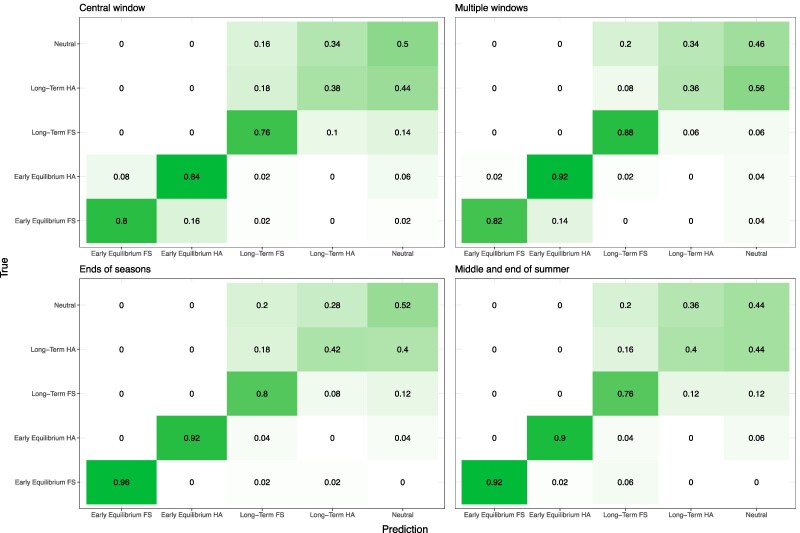
Confusion matrix of LDA performance. Confusion matrices show LDA model performance in classifying genetic signals associated with fluctuating selection (FS; *s* = 0.5) or heterozygote advantage (HA; *s* = 0.1) relative to one another or neutrally evolving loci. The predicted selection type and sampling point is labeled on the *x*-axis with the model's true class on the *y*-axis. The proportion of calls for each combination is shown within the respective tile.

Our results are promising, with high classification accuracy even being observed for scenarios where heterozygote advantage and fluctuating selection produce the most similar SFS- and haplotype-based statistics (i.e. *s* = 0.1 for the former and *s* = 0.5 for the later; Fig. 7). For these LDAs, we find that combining samples taken at multiple seasonal time points leads to the most accurate distinction of fluctuating selection at early equilibrium, with adaptive fluctuating loci sampled at the end of each season being correctly classified in 96% of cases, with slightly worse results (92%) when using data from the middle and end of summer. In contrast, using a single time point and multiple windows had the highest accuracy when predicting fluctuating selection at long-term equilibrium, though accuracy was lower with 88% of cases being correctly classified with the remaining 12% equally wrongly classified as either long-term heterozygote advantage or neutral evolution. This is potentially due to the signal of selection at this timepoint becoming so narrow that it cannot perturb the 10 kb window over which statistics are calculated and thus does not appear statistically distinct from neutrality (Figs. S10 to S12). Neutral simulations are incorrectly classified as long-term fluctuating selection in only 6% of cases, whereas long-term fluctuating selection is misclassified as neutral in 20% of cases. Overall, this indicates that the genomic signal of long-term fluctuating selection is distinguishable from neutrality in most scenarios (Fig. 7).

Examining results from fluctuating selection simulated at all different strengths showed that the sampling at the end of each season had the highest accuracy for strong and weak early equilibrium fluctuating selection (76% when *s* = 0.1, and 100% when *s* = 1; Figs. S21 and S22). The end of season approach is also the best option for weak long-term fluctuating selection (s = 0.1, accuracy = 14%; Fig. S21), however for strong long-term fluctuating selection the multiwindow approach had the greatest accuracy (100%; Fig. S22). When the highest classification accuracy was averaged across all strengths of selection to determine the best method for overall discrimination of selection, the multiple window approach was the most accurate (an average 67% accuracy for all selection types and strengths; Fig. 7, Figs. S21 and S22). Garud's H1, H123, H2/H1, and nucleotide diversity formed the basis of every LDA model tested in our analysis, with all but one also utilizing the variance of the SFS. Together, these results demonstrate that combining different summary statistics and utilizing broad genomic and temporal (i.e. seasonal) sampling can substantially improve power to distinguish fluctuating selection from other forms of selection compared with considering only individual statistics.

## Discussion

Multiple theoretical and empirical investigations of fluctuating selection have emerged over the past decades (Johnson et al. 2023), signaling the growing interest of this still poorly studied mode of selection among population genetic researchers. Previous studies have largely examined allele frequency trajectories over time, with the authors being aware of a handful of studies that focus on the genomic impact of fluctuating selection on linked neutral diversity (Barton 2000; Huerta-Sanchez et al. 2008; Taylor 2013; Wittmann et al. 2023). This study represents a major expansion of our current understanding of the population genetic signals of fluctuating selection, being the first to evaluate the performance of statistics that leverage haplotype information and methods developed to identify balancing selection. In addition, we use LDA to identify the signals of fluctuating selection from data sampled at one or two timepoints across a single season. Moreover, most previous studies of fluctuating selection have focused on scenarios involving random allelic fluctuations (Haldane and Jayakar 1963; Takahata et al. 1975; Gillespie 1997; Barton 2000; Huerta-Sanchez et al. 2008; Taylor 2013) whereas this study focuses on regular fluctuations caused by seasonally varying selection pressures, reflecting the mounting empirical evidence for this phenomenon in *Drosophila* and other species (Bergland et al. 2014; Machado et al. 2021; Behrman and Schmidt 2022; Pfenninger and Foucault 2022; Rudman et al. 2022; Bitter et al. 2024; Nunez et al. 2024) as well as the emergence of plausible mechanistic models (Wittmann et al. 2017, 2023; Bertram and Masel 2019; Park and Kim 2019; Kim 2023).

A recent study of fluctuating selection that used the same oscillatory model employed here, found that linked neutral diversity generally decreases around adaptive fluctuating loci (Wittmann et al. 2023), though closely linked variation tends to slowly increase after the start of selection. This results in a distinctive genomic pattern characterized by a sharp peak centered within a broader depression of neutral diversity, which is distinct from the genetic patterns generated by either positive or balancing selection. This characteristic fluctuating selection signature was only partly captured in the present study, with samples taken at early equilibrium showing substantial decreases in neutral variation linked to the selected site, though neutral diversity at our central 10 kb window never significantly exceeded expected levels even when sampling occurred at long-term equilibrium (>97,000 generations; Fig. 2). Additionally, varying the strength of the reversal of dominance (*h* > 0.5) did not cause any qualitatively different behaviors in the allele frequency trajectories, nor the population genetic patterns surrounding the fluctuating locus (Figs. S1 and S2). Notably, Wittmann et al. state that the width of the diversity peak around the selected site only appears below a genetic distance of 0.01 cM, which only amounts to 100 bp under the scaled recombination rate used for the simulations in our study. Accordingly, this peak is unlikely to be evident in the 10 kb windows used in the present study, confirming Wittmann and colleagues' comment that this signature may be difficult to identify in empirical data. Our results offer a conservative estimate of the effects of fluctuating selection on linked neutral genetic variation, as the observed signals are larger and more pronounced in simulations with a higher population (see Appendix 2).

Our results also share similarities with the reported effects of alternate selection scenarios with similar features to fluctuating selection, including a generalized model of recurrent partial sweeps (i.e. when selection pressures change or weaken prior to fixation of selected variants; Coop and Ralph 2012). In this model, the SFS becomes increasingly skewed towards rare variants and away from intermediate variants as the rate of recurrent sweeps increases, with high frequency alleles also increasing but tending to become fixed with subsequent sweeps (Coop and Ralph 2012). Adaptive fluctuating loci produce similar SFS distortions, with decreases in intermediate frequency variants and increased numbers of high frequency variants, though we observe an additional loss of singletons that is distinct from the recurrent partial sweep scenario (Fig. S5). Similar patterns have also been observed in a model of randomly fluctuating adaptive loci reported by Huerta-Sanchez and colleagues (Huerta-Sanchez et al. 2008), with randomly fluctuating loci also distorting the SFS by reducing intermediate frequency variants in favor of more variants at high frequencies.

In general, early fluctuating selection tends to alter the second to fourth moments of unfolded SFS in the same direction as positive selection, with both types of selection causing higher variance and decreased skew and kurtosis relative to neutrally evolving loci, though the magnitude is much greater for positively selected loci. These differences appear to be driven by the complete removal of intermediate variants from the SFS by positive selection, while these variants remain present at low levels under fluctuating selection (Fig. S5). As fluctuating selection continues, the SFS converges on that expected of classical balancing selection where we observe the maintenance of intermediate variants. The use of the balancing selection statistic NCD demonstrates this clearly, where we see the differences between fluctuating selection and heterozygote advantage become nonsignificant long-term. At the same time, haplotype-based statistics become distinct for stronger selection coefficients (*s* ≥ 0.5) maintaining the possibility of distinguishing these two forms of balancing selection when selection is strong. These signatures of fluctuating selection persevere with the incorporation of background selection, with only a minor reduction in the width of the signal observed around the fluctuating locus (Fig. S23).

A key finding in the present study is that the population genetic signals created by fluctuating selection depend upon the time that genomic data was collected within the seasonal cycle for many statistics. This seasonal dependence was also seen in Wittmann and colleagues' investigation of fluctuating selection signatures at long-term equilibrium (Wittmann et al. 2023), where they demonstrate that the sharp neutral diversity peak surrounding the selected site becomes most pronounced in the middle of the season, though effects further from the selected site are constant across the seasonal cycle. While we also observe constant diversity values across both seasons for fluctuating selection at long-term equilibrium (Fig. 4), our most extreme statistical values occurred at the season's end rather than the mid-season maximum observed by Wittmann and colleagues. These differences appear to be due to the two seasonal alleles appearing at 50% frequency at the onset of selection in in the Wittman et al. study, meaning that both alleles are effectively unlinked to their backgrounds, whereas fluctuating selection in the present study commences after the introduction of a de novo beneficial mutation that is strongly linked to background neutral variation. The latter scenario produces a partial sweep pattern that persists into early equilibrium, whereas a much smaller decline in neutral diversity is observed around the selected site following the onset of selection in the Wittman et al. study, which eventually grows to exceed neutral diversity levels over time (Wittmann et al. 2023).

Another important finding in our study is that the seasonal dependence of fluctuating selection signatures is a generic feature of the testing statistics, and detecting adaptive fluctuating loci is optimized by sampling at the end of season associated with the de novo beneficial allele. Our Linear Discriminant Analyses further highlight that this seasonal dependence can help to discriminate fluctuating selection from other modes of selection that lack such temporal dependencies, with substantial power gains being observed when samples from different seasonal time points were used for classification. Moreover, recording signals that peak toward the end of one season before becoming largely absent at the end of the following season may provide evidence for the favored season of each allele as well as identify the derived allele.

Another advantage of leveraging multiple statistics is that we are able to distinguish between fluctuating selection and heterozygote advantage at all tested selection coefficients, whereas fluctuating selection acting at weaker strengths (*s* ≤ 0.1; unscaled *s* ≤ 0.001) cannot be distinguished from neutral evolution for most individual statistics. However, estimates of selection strength of fluctuating alleles detected in empirical data range from 1% to 50% per generation (Bergland et al. 2014; Machado et al. 2021; Rudman et al. 2022; Bitter et al. 2024) suggesting that signals of fluctuating selection near the lower bound of empirical estimates should still be detectable. While our results suggest that the combination of multiple statistics may be sufficient to identify a considerable fraction of fluctuating loci from sequence data collected at a single time point, our results also emphasize that employing temporally varied sampling strategies promise to discriminate a much larger proportion of these loci.

## Conclusion

Our study confirms that fluctuating selection makes a distinctive impact on linked genetic variation that allows it to be distinguished from neutrally evolving regions and other modes of selection, including heterozygote advantage. Locus detection and discrimination from other modes of selection is particularly powerful when temporal information and genomic information from windows adjoining the selected site are leveraged. Nonetheless, it is difficult to deduce how the signatures observed in this study are affected by demographic and ecological forces experienced by natural populations that are not modeled in the present study. Accordingly, the field stands to benefit from more realistic simulation of fluctuating selection signals that add ecological and demographic realism—such bottlenecks, admixture, and seasonal population size changes—and that extend the model to include multilocus selection.

## Methods

### Simulations

Four forms of selection were simulated: positive selection in the form of soft and hard selective sweeps, balancing selection as heterozygote advantage, and seasonally fluctuating selection. Positive selection was simulated with the selection pressure acting at the first generation after the burn-in. For hard selective sweeps, a single mutation was added in the first generation at a frequency of 1/2*N_e_*. To establish multiple mutations for the soft selective sweep, an increased beneficial mutation rate was implemented to establish multiple beneficial mutations at the selected locus early in the simulation, imitating a multiple-origin soft sweep. Heterozygote advantage was simulated from a single mutation with the fitness of individuals dictated by the genotype at the selected site. Individuals with a heterozygous genotype at the selected site had a fitness of 1, and homozygote fitness being 1 minus the selection coefficient (*s*) of the alternate allele. Similarly, fluctuating selection was simulated from a single mutation in a binary two-season environment with the selection pressure implemented using a fitness model presented by Wittmann et al. (2023). The model comprises seasonal selection and dominance coefficients (*s* and *h*, respectively). Individual fitness depends on the genotype and the season (summer/winter), shown in Table 1. In the simulations, the selection and dominance coefficients were equal between seasons which were each 10 generations in length. Simulations of fluctuating selection with background selection followed the same approach as those of only a fluctuating locus with the addition of deleterious mutations that were allowed to occur across the simulated region in accordance with Huber et al. (2017) and Schrider (2020), with selection coefficients drawn from a *γ*-distribution and the DFE having a mean of −0.000133 and a shape of 0.35.

As models of fluctuating selection have been developed for *Drosophila melanogaster* populations, we use downscaled population parameters to increase the efficiency of the simulations while capturing the genetic patterns expected in natural populations of *Drosophila*. To simulate a population equivalent to a natural population with an effective population size of 1 million individuals, a recombination rate of 10^−8^ (Comeron et al. 2012), and a mutation rate of 10^−9^ (Schrider et al. 2013; Keightley et al. 2014), we use a down-scaled population size of 10,000, a recombination rate of 10^−6^ and mutation rate of 10^−7^. In accordance, the selection coefficient was also scaled such that *s* = 0.01 in a natural population corresponds to an s of 1 in the downscaled, simulated population. We do not scale season length, as this would result in seasons that are shorter than one generation, which cannot be represented in our simulation framework. However, we ensured that we capture seasonal oscillations in allele frequency across the season equivalent to those observed in empirical data. This emphasis on allele frequency amplitude allows us to conduct computationally feasible simulations while still capturing seasonal allele-frequency fluctuations. For these simulations, we maintained a constant population size across seasons. All simulations were of a 5 Mb segment with a selected locus at its center. Under the simulated recombination rate, this length is sufficient to capture the full extent of even the strongest expected signal—a hard selective sweep with *s* = 1. Importantly, this length also accommodates broader, long-term diversity signatures associated with fluctuating selection, as shown in Wittmann et al. (2023).

Each simulation replicate consisted of three parts, using an approach developed based on a recent benchmarking study of different simulation workflows in Johnson et al. (Johnson et al. 2024). Firstly, a coalescent burn-in was simulated using *msprime* (v. 1.2.0; Baumdicker et al. 2022) to establish neutral diversity and ensure coalescence of the lineages of the population in which selection was simulated. The tree sequence of the burn-in (Haller et al. 2019) was then read into the forward simulator, *SLiM* (v. 4.0.1; Haller and Messer 2023), as the starting population. The selected mutations were drawn into a genome in the starting population (as described above given the form of selection being simulated) and the simulation was run for 100,000 additional generations in a binary two-season environment. If the mutation was lost prior to the completion of the forward simulation, the simulation was restarted, reading in the coalescent burn-in data again. The number of restarts was recorded (Appendix 1). The tree sequence recording approach was used to sample 100 individuals for 60 consecutive generations (three seasonal cycles) at various time points throughout the simulation. This was done in *SLiM* by randomly sampling 100 individuals from the current population and saving them to the tree sequence data structure using the “treeSeqRememberIndividuals” function. Two key timepoints were included in the sampling: early equilibrium, when the selected locus first reaches a stable fluctuation; and long-term equilibrium (96,000 generations) when neutral diversity is expected to be in equilibrium using the 10*N_e_* criterion (Johnson et al. 2024) and being past the expected point of neutral equilibrium for linked neutral sites as determined by Wittmann et al (*4Np,* where *N* is the population size and *P* is the harmonic mean allele frequency; Wittmann et al. 2023). For fluctuating selection and heterozygote advantage, populations were also sampled after the allele frequency trajectory was at equilibrium for a whole seasonal cycle. For positive selection, early equilibrium was immediately after the selected allele became fixed in the population. This sampling scheme provided high temporal resolutions at some time points as well as the ability to watch the patterns of selection develop through time. The allele frequency of the selected allele was also recorded (Kelleher et al. 2016, 2018; Haller et al. 2019).

The final tree sequence data consisted of the coalescent burn-in as well as the forward-in-time simulation in which selection was acting. Neutral mutations were then overlaid onto the tree sequence data using msprime. The *pySLiM* (v. 1.0) and *tskit* (v. 0.5.2) libraries (Kelleher et al. 2018; Ralph et al. 2020; Baumdicker et al. 2022) were also used in the processing of the tree sequence throughout the simulation workflow.

### Calculation of Summary Statistics

Subsequent analysis of the tree sequence data was conducted using *tskit* (v. 0.5.2), *PySLiM* (1.0), and *scikit-allel* (v. 1.3.5; Miles et al. 2021). Statistics were calculated in 10 kb windows across the 5 Mb simulated segment. Tajima's D (Tajima 1989) and nucleotide diversity (Tajima 1983), and the number of segregating sites were calculated with *tskit*. Tajima's D was also calculated using *scikit-allel* (v. 1.3.5) which was used to calculate Watterson's theta (Watterson 1975), Garud's H1, H2, H2/H1, and H123 (Garud and Rosenberg 2015). In addition to these summary statistics, we also calculated the balancing selection statistic NCD (Bitarello et al. 2018). This statistic was calculated in Python, using eq. 1.


(1) $$\mathrm{NCD}=\sqrt{\frac{\sum_{i=1}^{n}{\left(\;p_{i}-\mathrm{TF}\right)}^{2}}{n}}$$

The NCD statistic uses the minor allele frequencies of mutations in a window and measures their squared distance to a target frequency (TF). We used three values for the TF, 0.5, 0.4 and 0.3. As NCD can be correlated to the number of segregating sites in the window, we standardized the NCD values by binning each window by the number of segregating sites. Bins increasing by increments of 25 were used. We then calculated the mean and standard deviation of NCD at each TF under neutral evolution. NCD was then standardized using eq. 2.


(2) $$\frac{\mathrm{NCD}-\mathrm{mea}n_{\mathrm{neutral}}}{sd_{\mathrm{neutral}}}$$

Variance, skew and kurtosis of the distribution of allele frequencies in each window were also captured. The variance was calculated with the Python *statistics* package, while skew and kurtosis were calculated using the *SciPy* package (v. 1.9.0; Virtanen et al. 2020). The SFS was calculated using *tskit*. The spectrums of 50 replicates for each selection type and sampling point were averaged. Haplotype frequencies were also calculated by counting the number of unique haplotypes for 50 replicates for each selection type and sampling point. The mean frequency of the 5 most common haplotypes for each selection time was calculated.

### Comparing Forms of Selection

Pairwise comparisons to obtain values of significance were calculated with a *t*-test using the “compare_means” function of *ggpubr* (v. 0.6.0) with the Hochberg multiple-testing correction. The different selection types were compared at the 10 kb window right on top of the selected site for each statistic investigated. Cohen's D was also calculated for each comparison (Cohen 1988; Grice and Barrett 2014) using the *lsr* R package (v. 0.5.2) to evaluate the overlap in distribution between different selection types for statistics that returned a significant *P*-value.

### Linear Discriminant Analysis

An additional 150 simulation replicates, only collecting data at the early equilibrium and long-term sampling points for neutral evolution, heterozygote advantage, and fluctuating selection, were conducted for the purpose of the LDA, leading to a total of 200 replicates. LDA was conducted using a stepwise approach, by first determining which statistics separate the groups best and using this tailored subset to determine the formula for the LDA. Analysis was conducted without positive selection to ensure the model was tailored to distinguishing early equilibrium and long-term heterozygote advantage and fluctuating selection, as these types of selection show highly similar distributions for many individual statistics. We used four approaches; the first used only the values of each statistic at the central window from the final generation of summer at the relevant sampling point in the simulation. The second utilized values from the central window, the adjacent window and a window 250 kb away from the selected site for each statistic. The third used only the central windows but values sampled from the end of each season in the relevant seasonal cycles, and the final approach used values from the central window sampled at the middle and end of summer. The model was developed and trained on 150 (out of 200) replicates and then tested on the remaining 50 replicates. The “greedy.wilks” function, from the *r* package *klaR* (v. 1.7), was run over all the statistics to determine which set of variables best separated the different selection types. The level for the F-test decision (‘*niveau*’ argument of function) was set to 0.05 (i.e. the maximum *P*-value of difference between the previous model and the model with the additional variable). This function conducts a stepwise forward variable selection, testing different combinations of variables to determine the set that confers the most separation and returns a set of variables for subsequent LDA analysis. In some cases, the variables were found to be collinear—when this occurred it was found that if NCD (TF = 0.5) was removed from the stepwise analysis, a model could be generated and the LDA performed. Details of these cases can be found in the supplementary tables (Table S1). The LDA was conducted using the *r* package *MASS* and its “lda” function. The model was first trained on the 150 training data replicates. It was then evaluated on the remaining 50 replicates using a cross-validation approach and the accuracy of prediction of the model for each type and sampling point of selection was calculated and recorded (Fig. 7, Figs. S21 and S22). The mean accuracy for each approach was calculated by averaging the correct prediction values for each selection type and sampling point.

## Supplementary Material

evag082_Supplementary_Data

## Data Availability

The code to replicate the simulations, data processing, and calculation of summary statistics can be found at https://github.com/olivia-johnson/FootprintsofFluctuatingSelection.
