# Supplementary material for: Genetic Footprints of Seasonal Fluctuating Selection: A Comparison With Established Selection Forms
Source: Genome Biol Evol. 2026 Apr 2;18(4):evag082. doi: 10.1093/gbe/evag082 (PMC13092345; doi:10.1093/gbe/evag082)
Supplement: evag082_Supplementary_Data [file evag082_supplementary_data.zip › SIFinal.pdf]

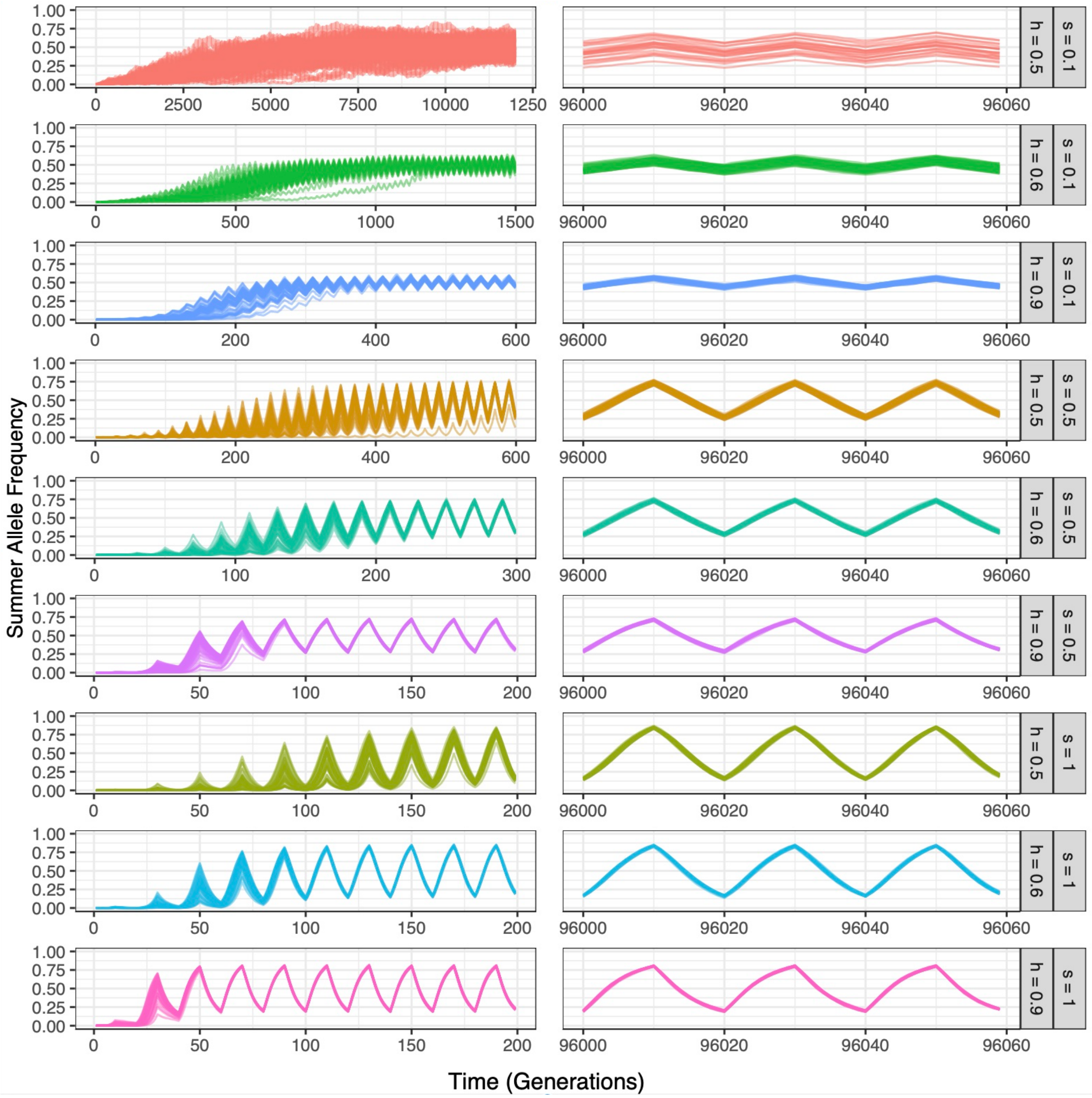

Figure S1. Allele frequency trajectories at Early Equilibrium and Long-Term Fluctuating selection for dominance coefficients of 0.5, 0.6, and 0.9.

Frequency of the summer-favoured allele across time, shown for three different selection coefficients (*s*; 0.1, 0.5, 1) and two dominance coefficients (*h*; 0.5, 0.6; 0.9) (labels on the right-hand side). Starting from a single mutation, the summer-favoured allele gradually increases and both alleles oscillate around a mean frequency that eventually reaches a stable equilibrium, with alleles that have a higher dominance and stronger selection reaching their equilibrium value more quickly (left hand panels) and showing more consistent oscillatory behavior and lower stochastic allele frequency variance at equilibrium after 96,000 generations (right hand panels). 50 replicates are depicted in each panel.

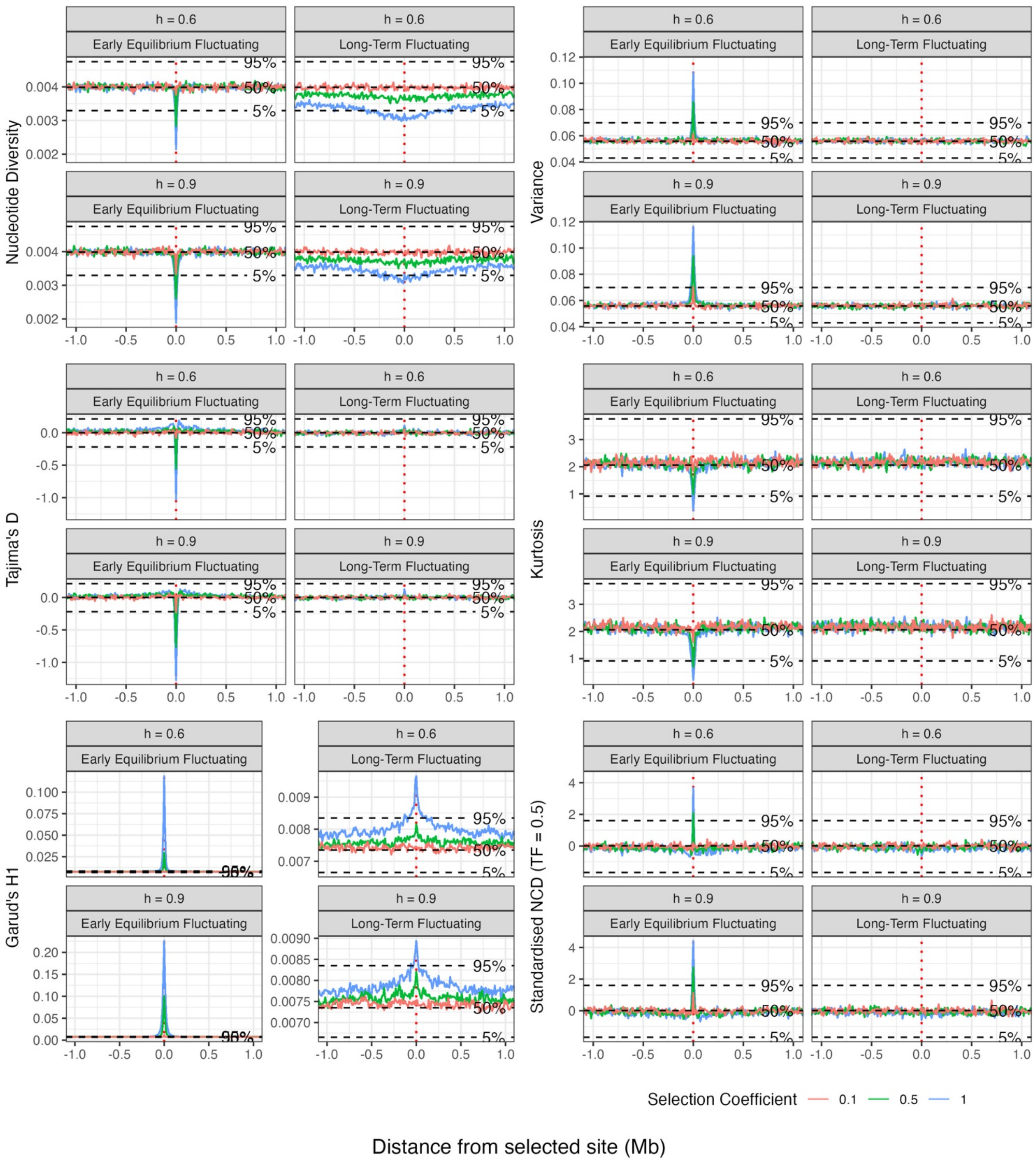

Figure S2. Distribution of population genetic statistics for Early Equilibrium and Long-Term Fluctuating selection for non-additive dominance coefficients of 0.6 and 0.9

Statistics were calculated in 10kb windows across the simulated region and were averaged across 50 replicates for three selection coefficients: 1; 0.5; and 0.1, (see key). The vertical dashed red line signifies the position of the selected site. Horizontal dashed black lines illustrate the 5%, 50% and 95% quantiles of the 50 replicate values of each statistic for neutral simulations (denoted by label).

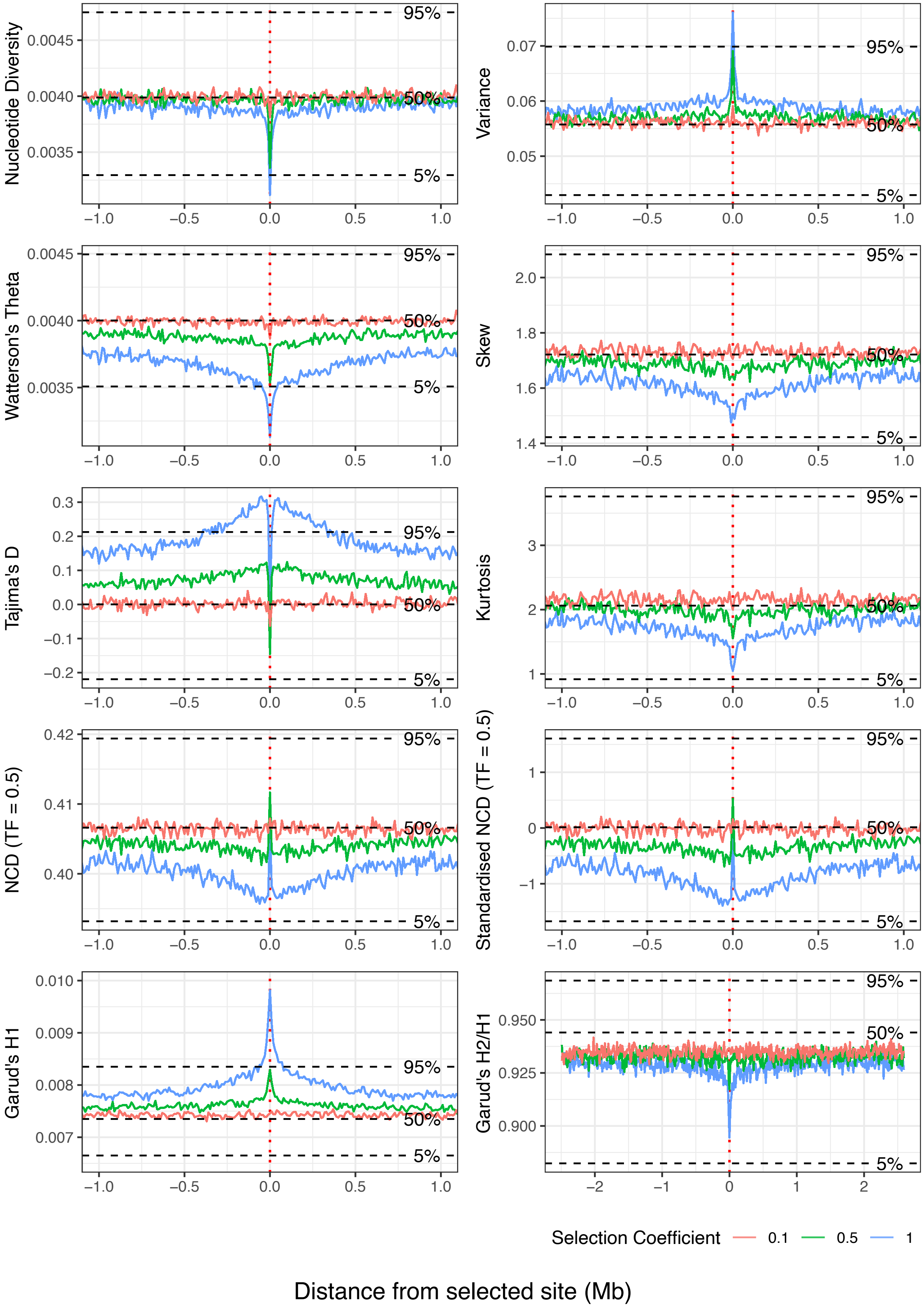

Figure S3. Signatures of early fluctuating selection sampled at generation 2570.

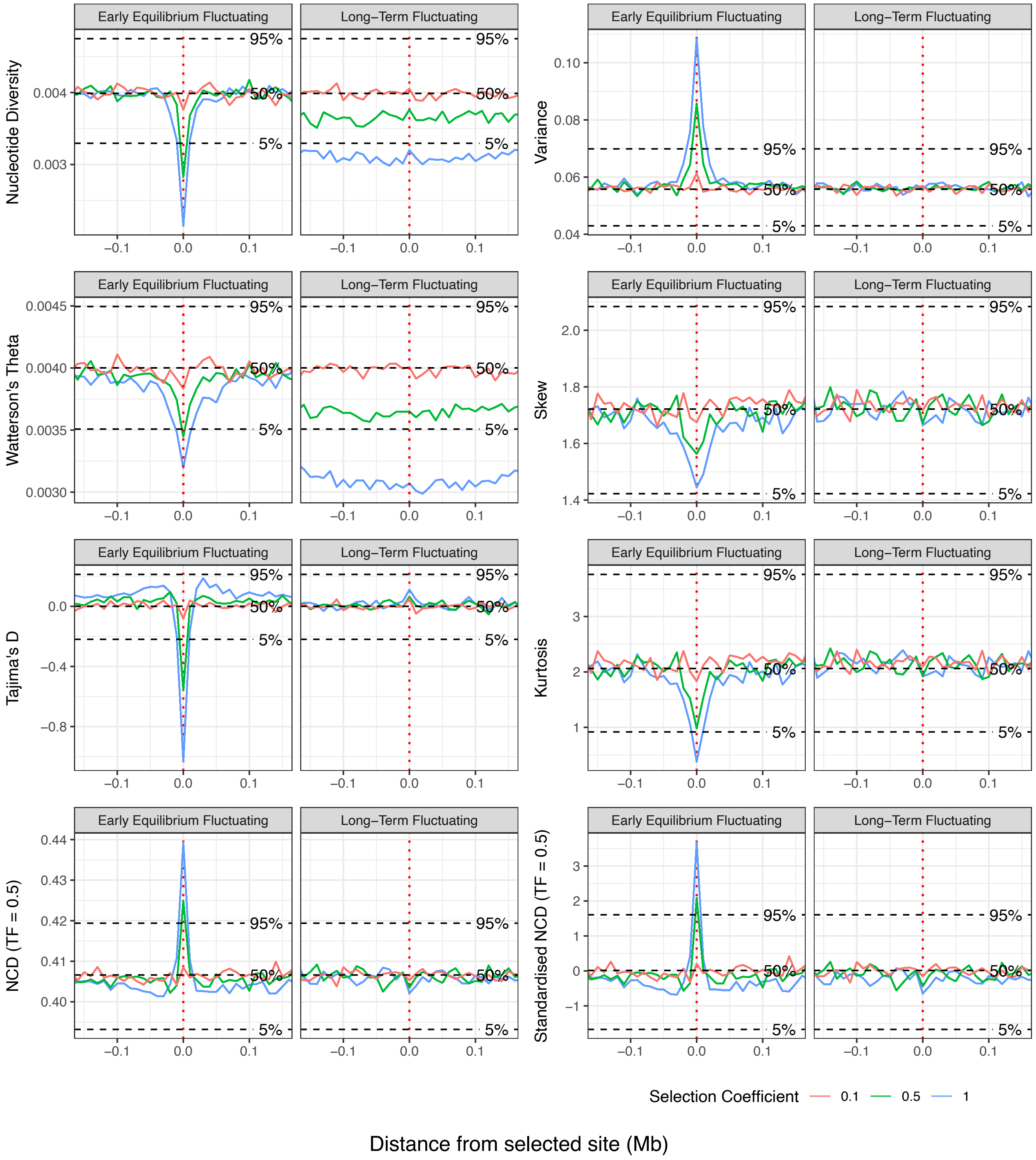

Figure S4. Footprints of fluctuating selection across time immediately surrounding the selected site. The results depicted in Figure 2 enhanced to visualize the signal immediately surrounding the selected site.

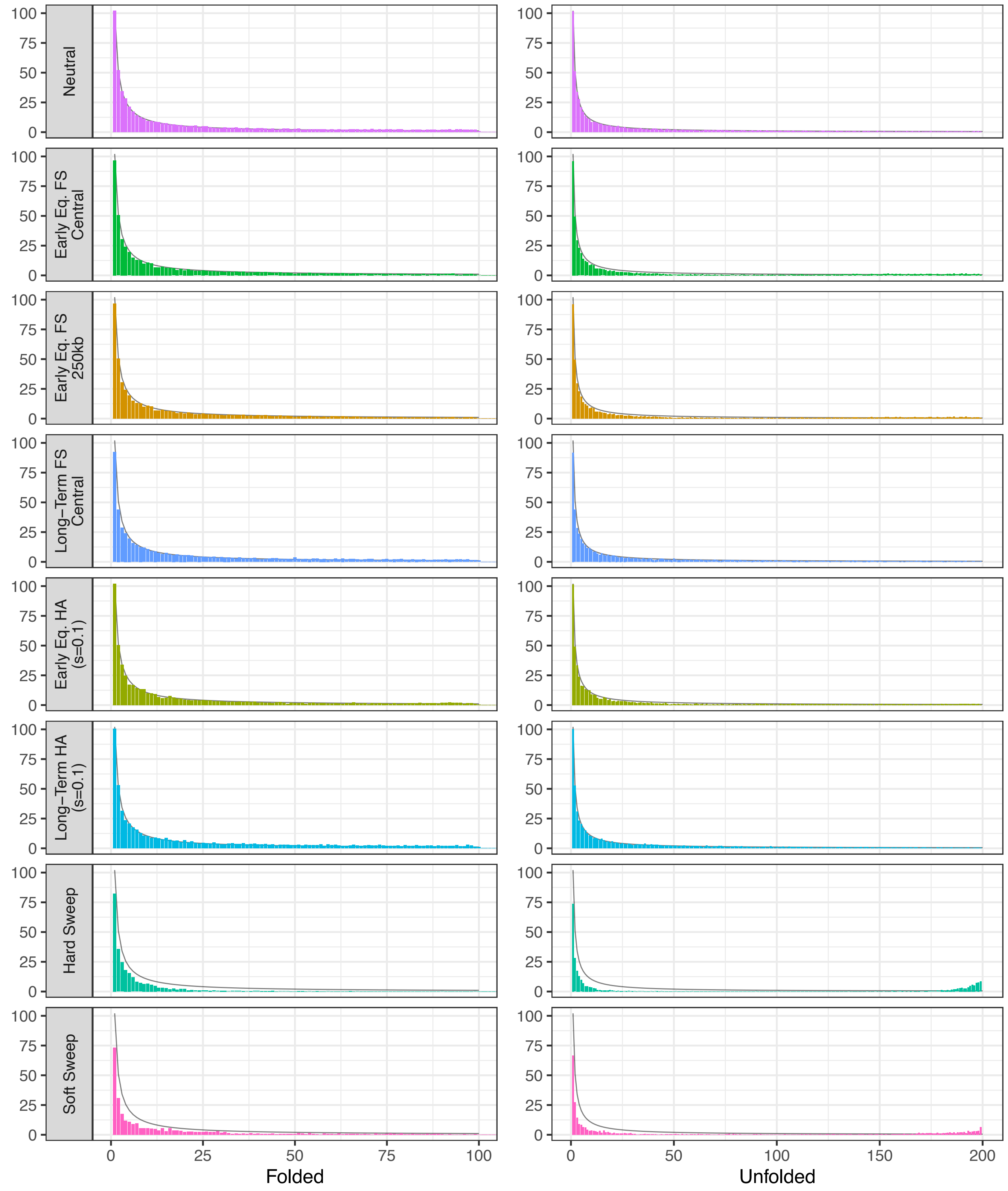

Figure S5. Folded and unfolded site frequency spectrum for each selection type.

Neutral evolution, early equilibrium fluctuating selection (FS) at the central window and at a window 250kb from the selected site, long-term fluctuating selection, hard and soft positive selection after fixation (all $s = 0.5$), and early equilibrium and long-term heterozygote advantage (HA; $s = 0.1$) all at the central window.

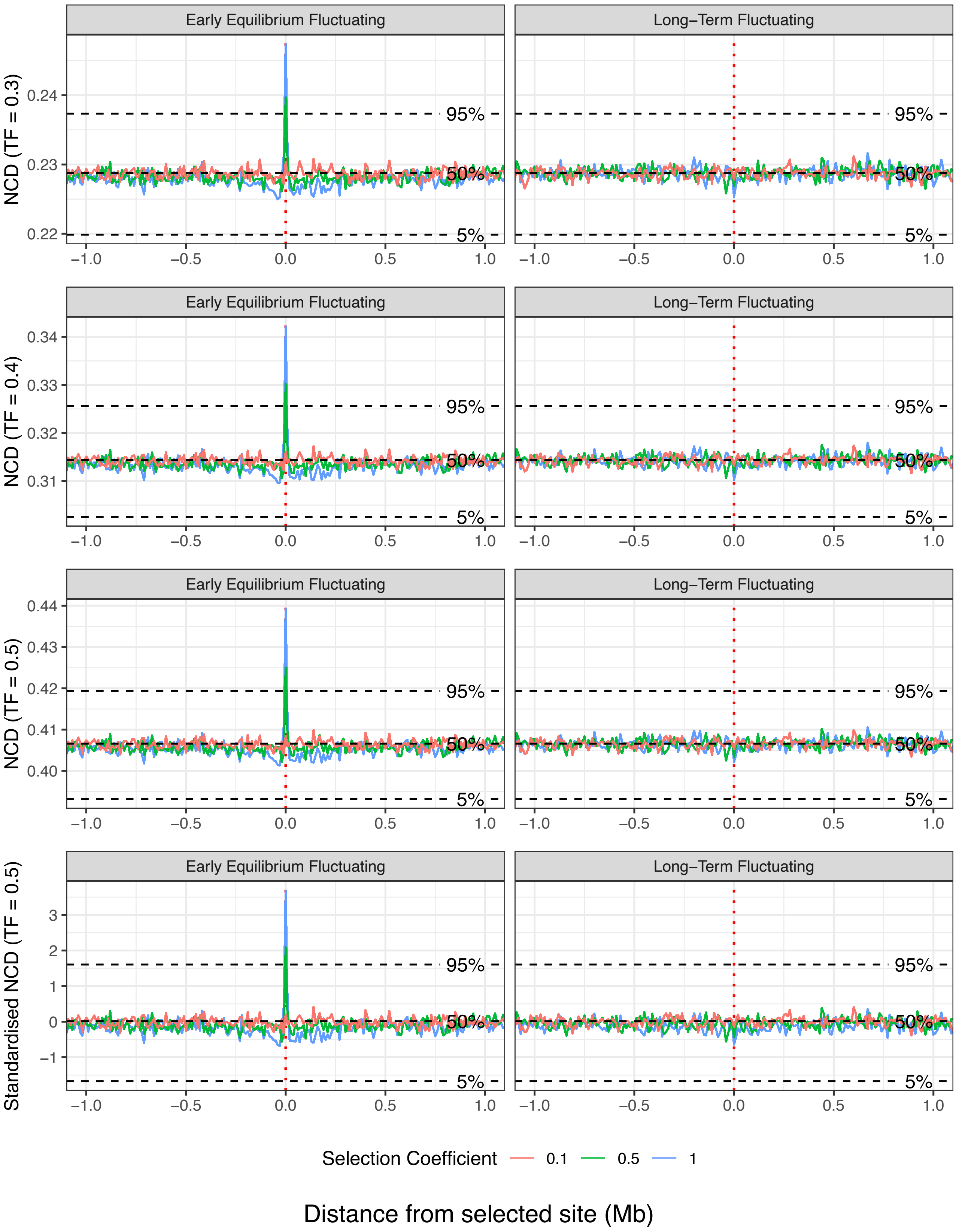

Figure S6. Signatures of fluctuating selection in NCD for all target frequencies.

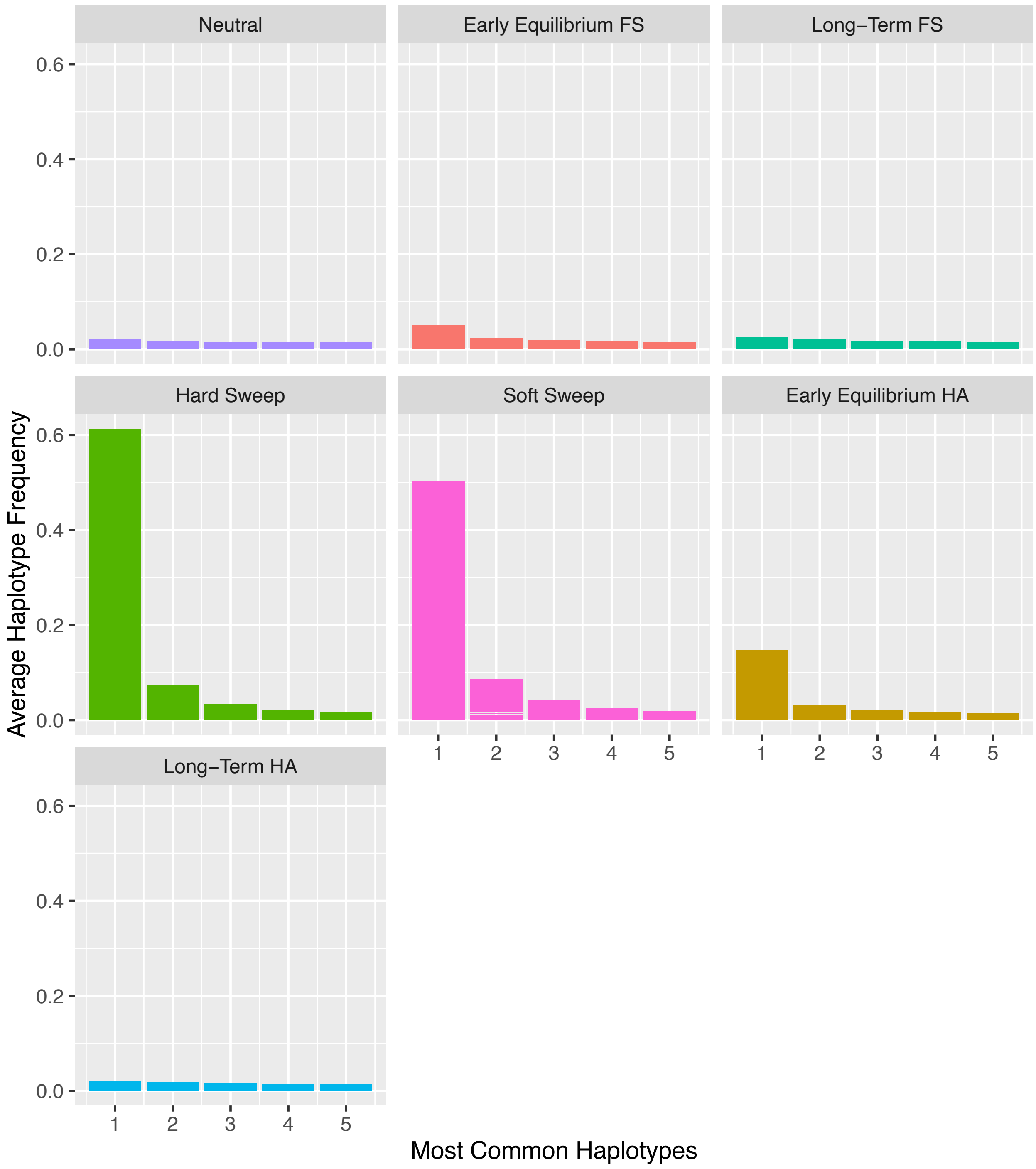

Figure S7. The frequency of most common haplotypes when *s* = 0.5 for fluctuating (FS) and positive selection (hard and soft sweeps) and 0.1 for heterozygote advantage (HA).

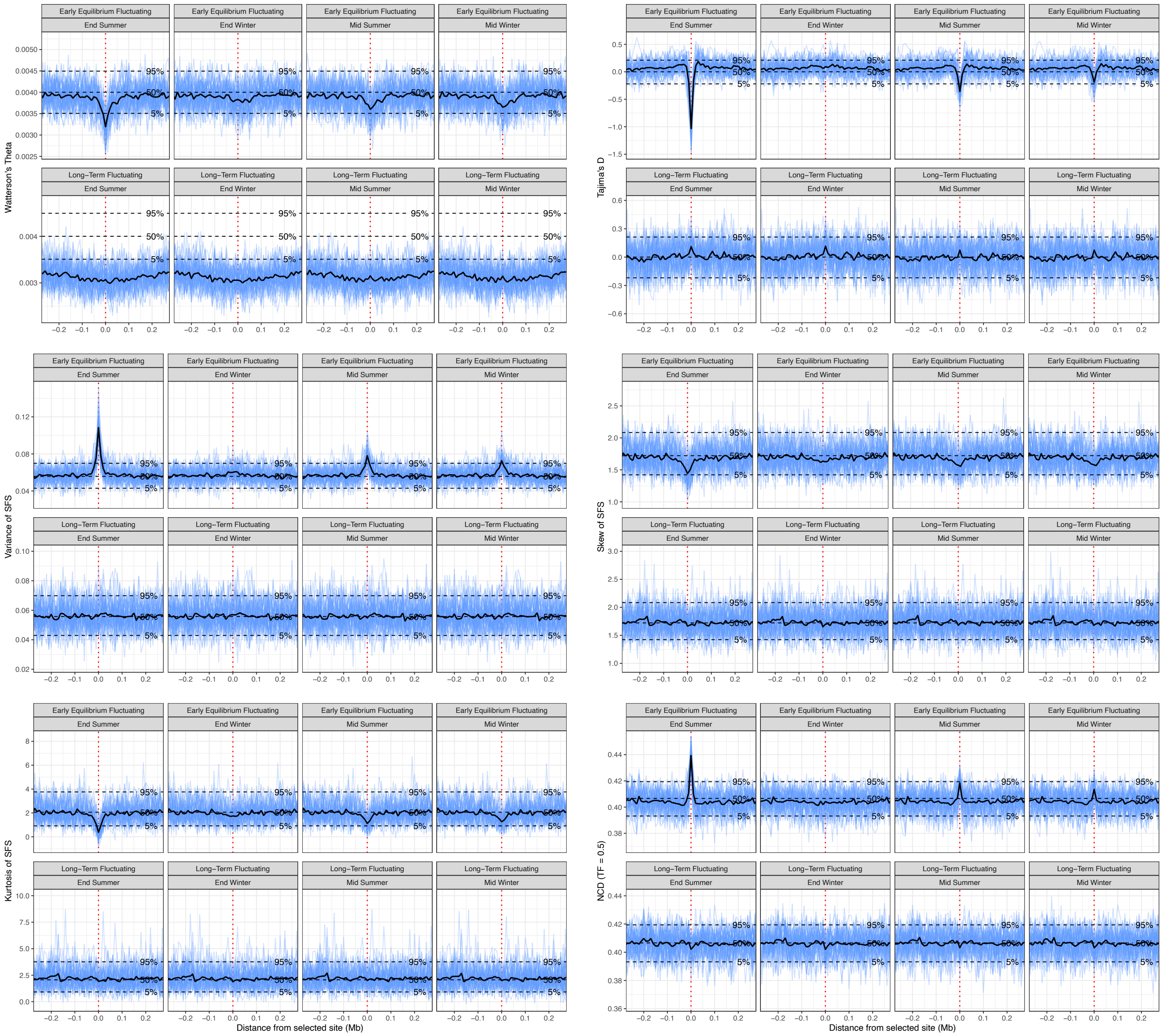

Figure S8. The changes in the signature of fluctuating selection across the seasonal cycle.

Signature of fluctuating selection in Watterson's theta, Tajima's D, the moments of the SFS and NCD for early equilibrium and long-term fluctuating selection are plotted at the middle and end of summer and winter across a single seasonal cycle. Data simulated with a selection coefficient of 1 is shown as it gives the clearest patterns, which are less pronounced as the selection coefficient decreases. Horizontal dashed black lines illustrate the 5%, 50% and 95% quantiles of the 50 replicate values of each statistic for neutral simulations (denoted by label).

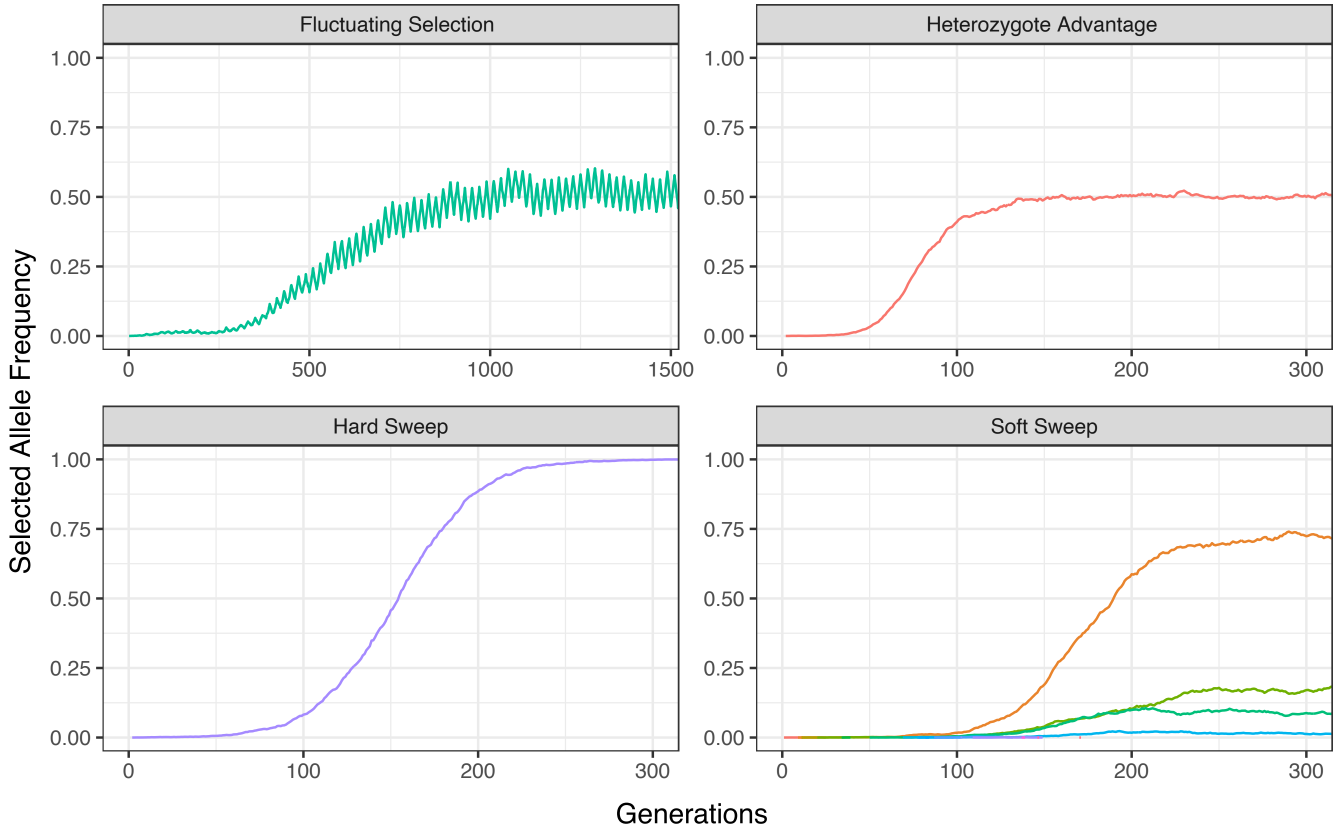

Figure S9. Allele frequency trajectory of a single replicate for each form of selection. Time in simulated generations is shown along the x-axis and the allele frequency of the selected allele is shown on the y-axis. All simulations had a selection coefficient of 0.1 and the data from one replicate is shown on each panel. The establishment of a selected allele/trait is shown for each form of selection. Balancing selection as heterozygote advantage and fluctuating selection are shown until the allele has reached a stable equilibrium. For fluctuating selection, this follows the summer-favored allele. For soft sweeps, each beneficial mutation conferring the selected trait is coloured differently, and the combined sum of the frequencies of each mutation gives the frequency of the trait. Hard and soft sweeps are shown until just after fixation.

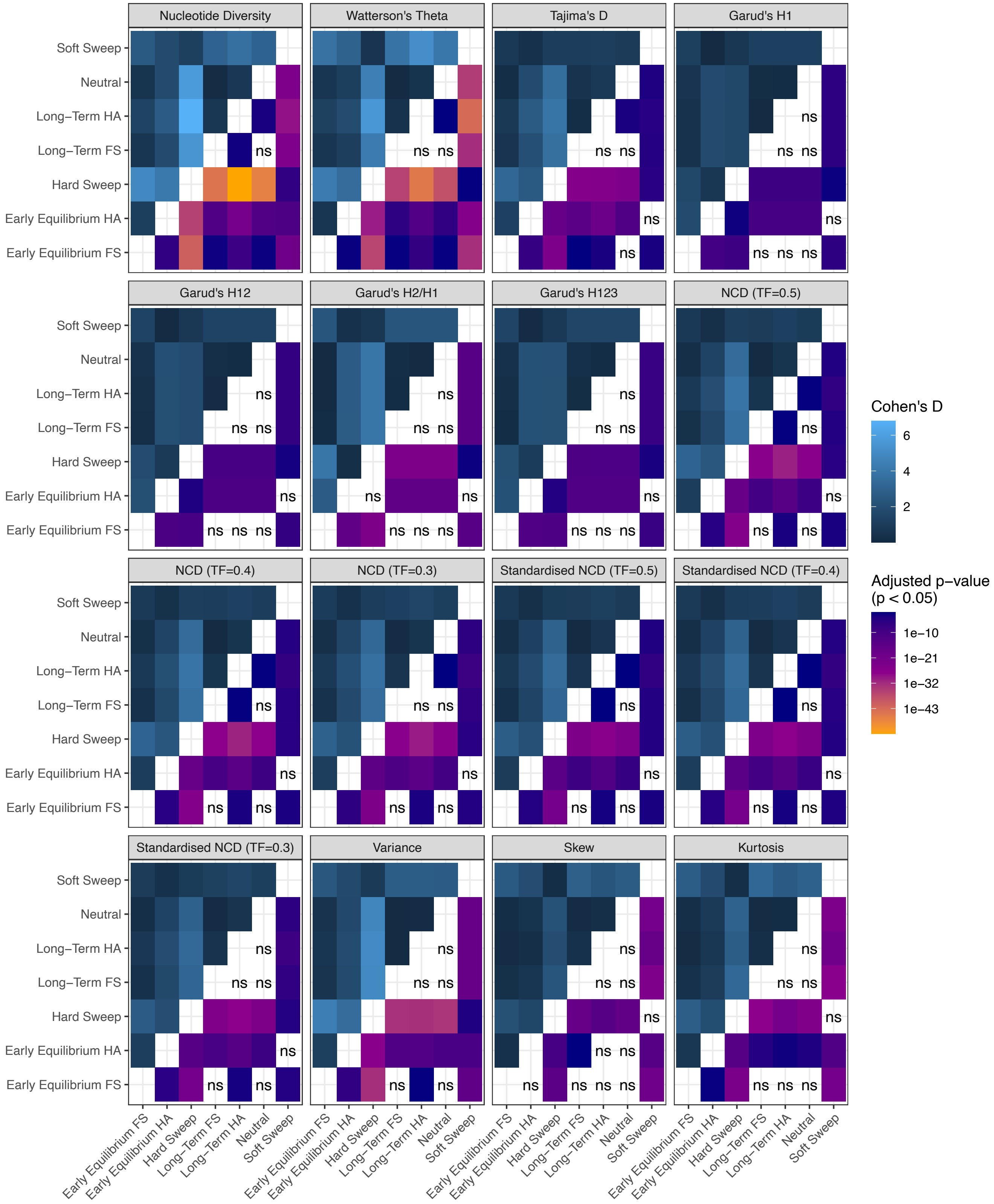

Figure S10. Heatmap of *p*-value and Cohen's D for pairwise comparisons between heterozygote advantage (HA), fluctuating (FS) and positive selection with a selection coefficient of 0.1.

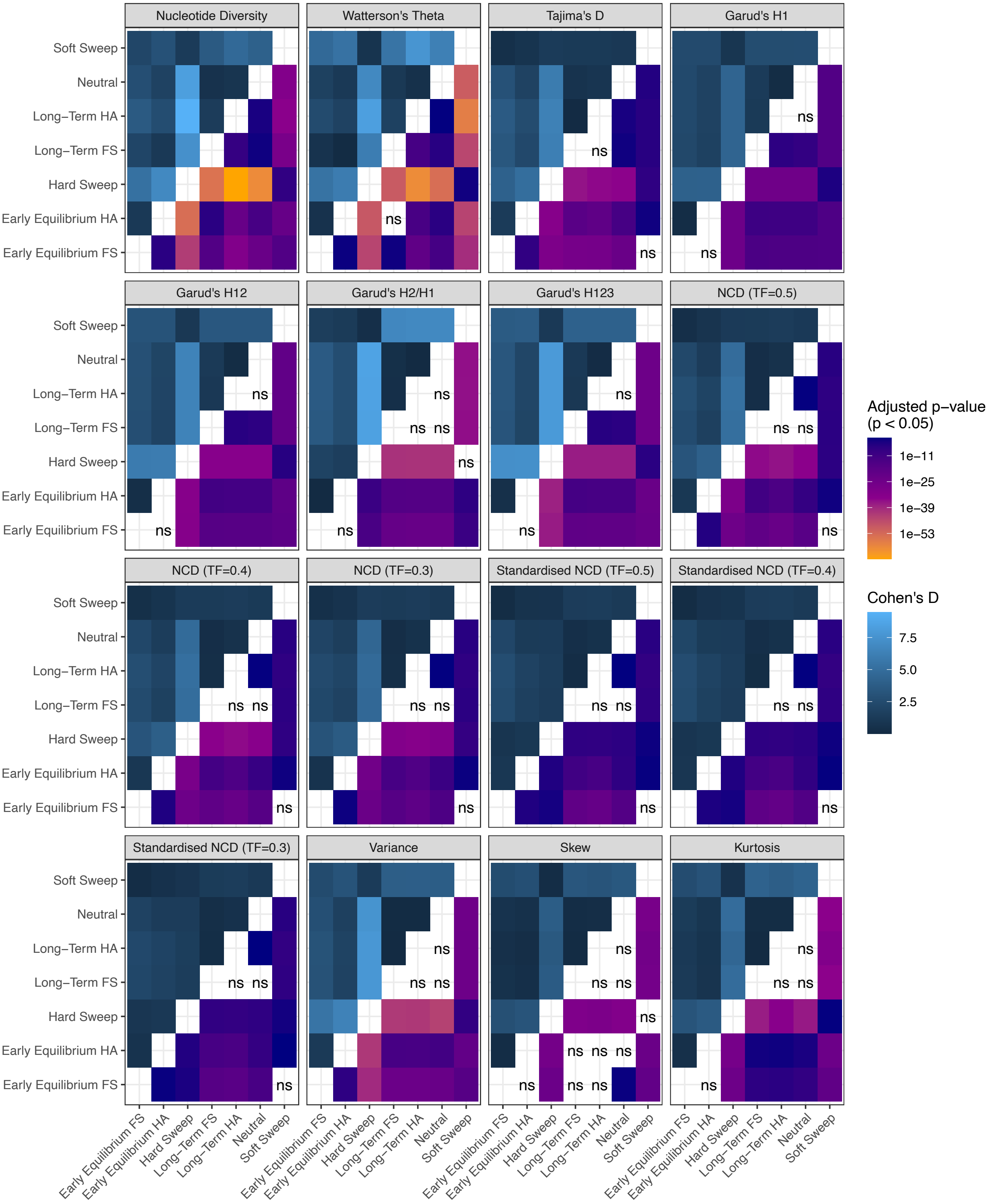

Figure S11. Heatmap of *p*-value and Cohen's D for pairwise comparisons between heterozygote advantage (HA) with a selection coefficient of 0.1, and fluctuating selection (FS), and positive selection with a selection coefficient of 0.5.

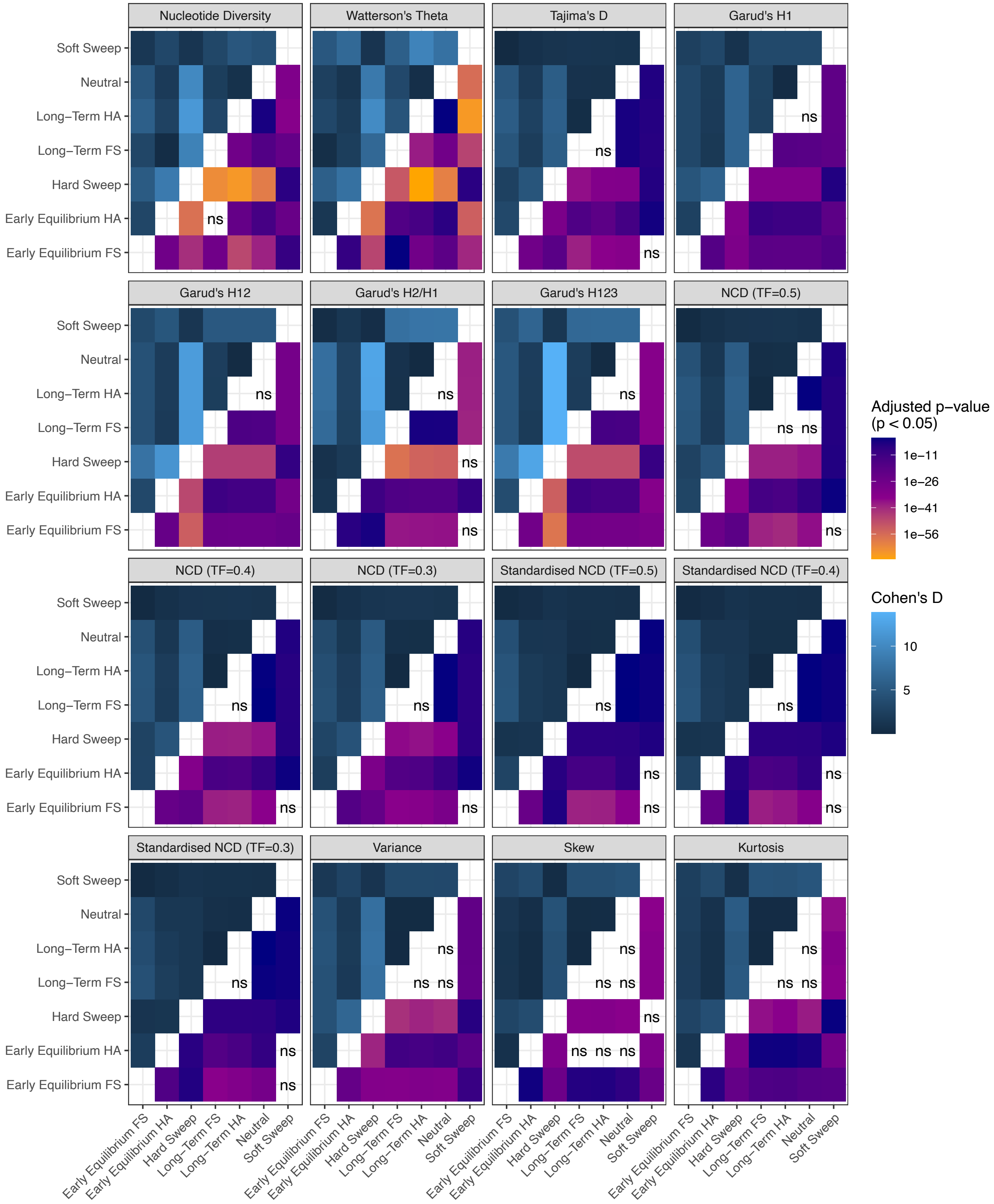

Figure S12. Heatmap of *p*-value and Cohen's D for pairwise comparisons between heterozygote advantage (HA) with a selection coefficient of 0.1, and fluctuating selection (FS), and positive selection with a selection coefficient of 1.

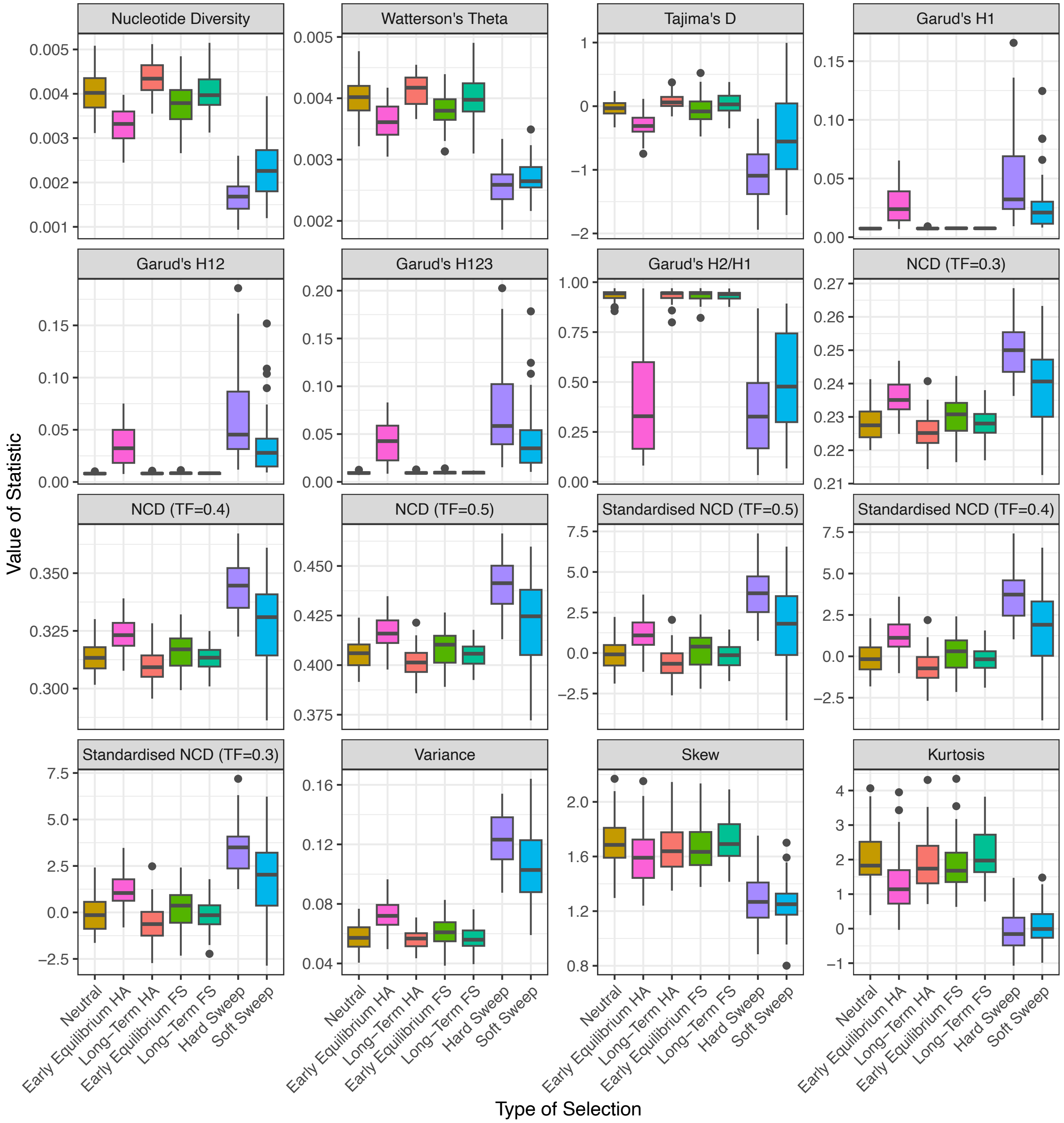

Figure S13. Population genetic statistics at the 10 kb window centered over the selected site for positive selection, fluctuating selection (FS), and heterozygote advantage (HA) with *s* = 0.1.

Boxplots showing the distribution of values for different statistics, labelled at the top of each facet, evaluated for four modes of selection (see x-axis labels; 50 replicates and *s* = 0.5 for each selection coefficient excluding heterozygote advantage where *s* = 0.1). Positive selection in the form of hard (purple) and soft (blue) selective sweeps often cluster together and are distinct from Early Equilibrium and Long-Term Heterozygote Advantage (HA; pink and red) and Fluctuating Selection (FS; green and turquoise) and neutral evolution (gold) for most statistics.

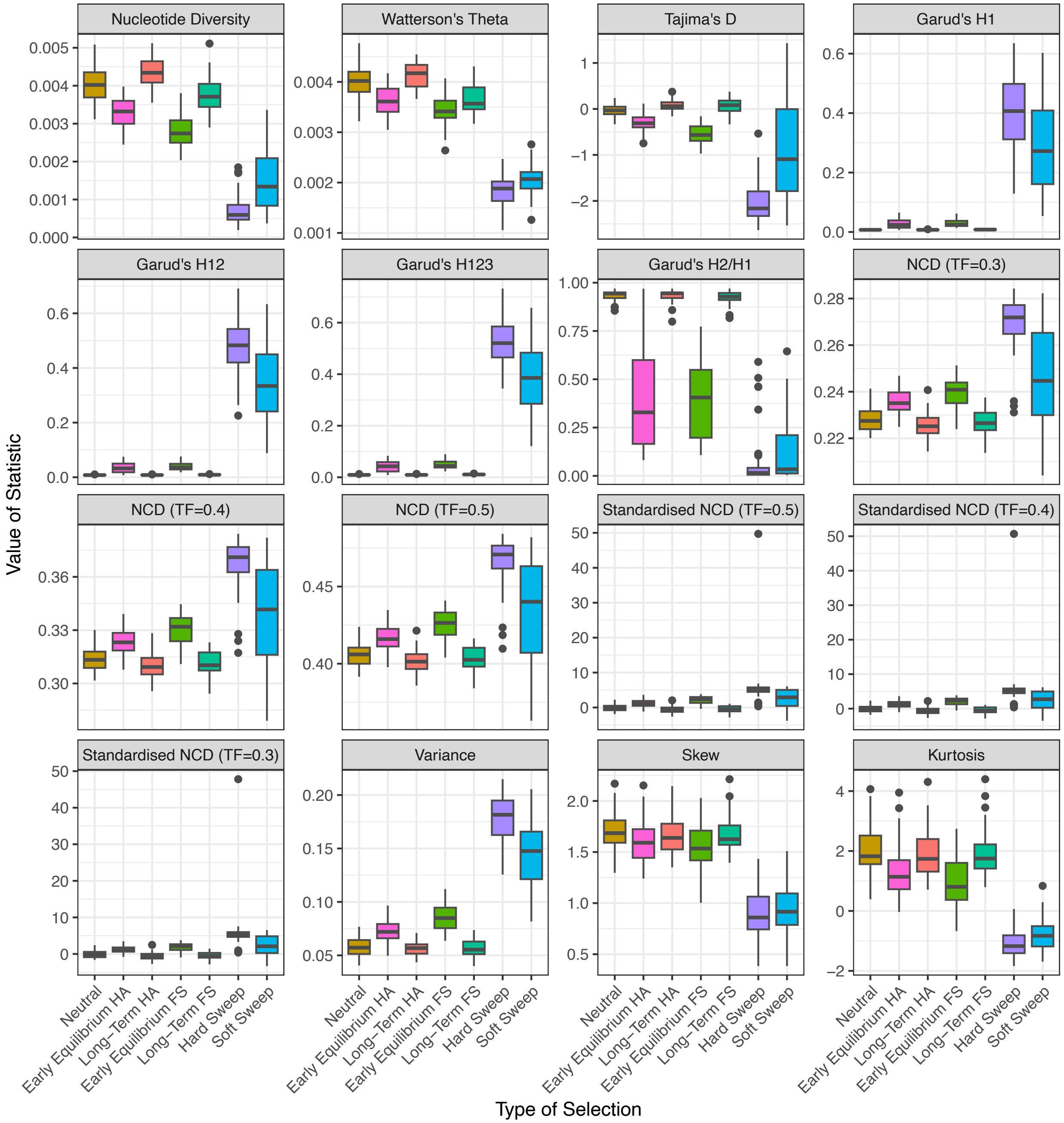

Figure S14. Population genetic statistics at the 10kb window centered over the selected site for different modes of selection.

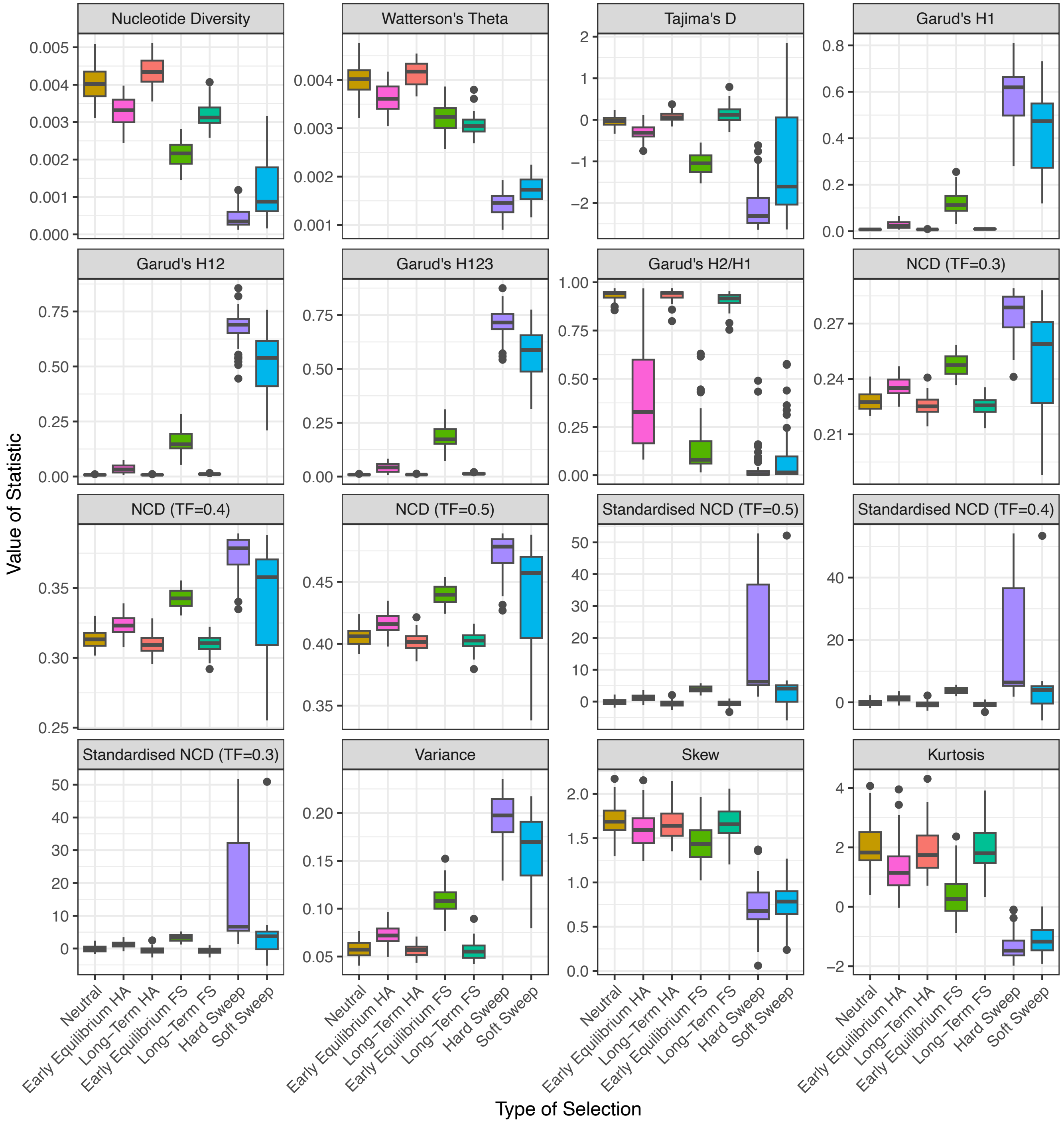

Figure S15. Population genetic statistics at the 10 kb window centered over the selected site for positive selection and fluctuating selection (FS) (*s* = 1) with *s* = 0.1 for heterozygote advantage (HA).

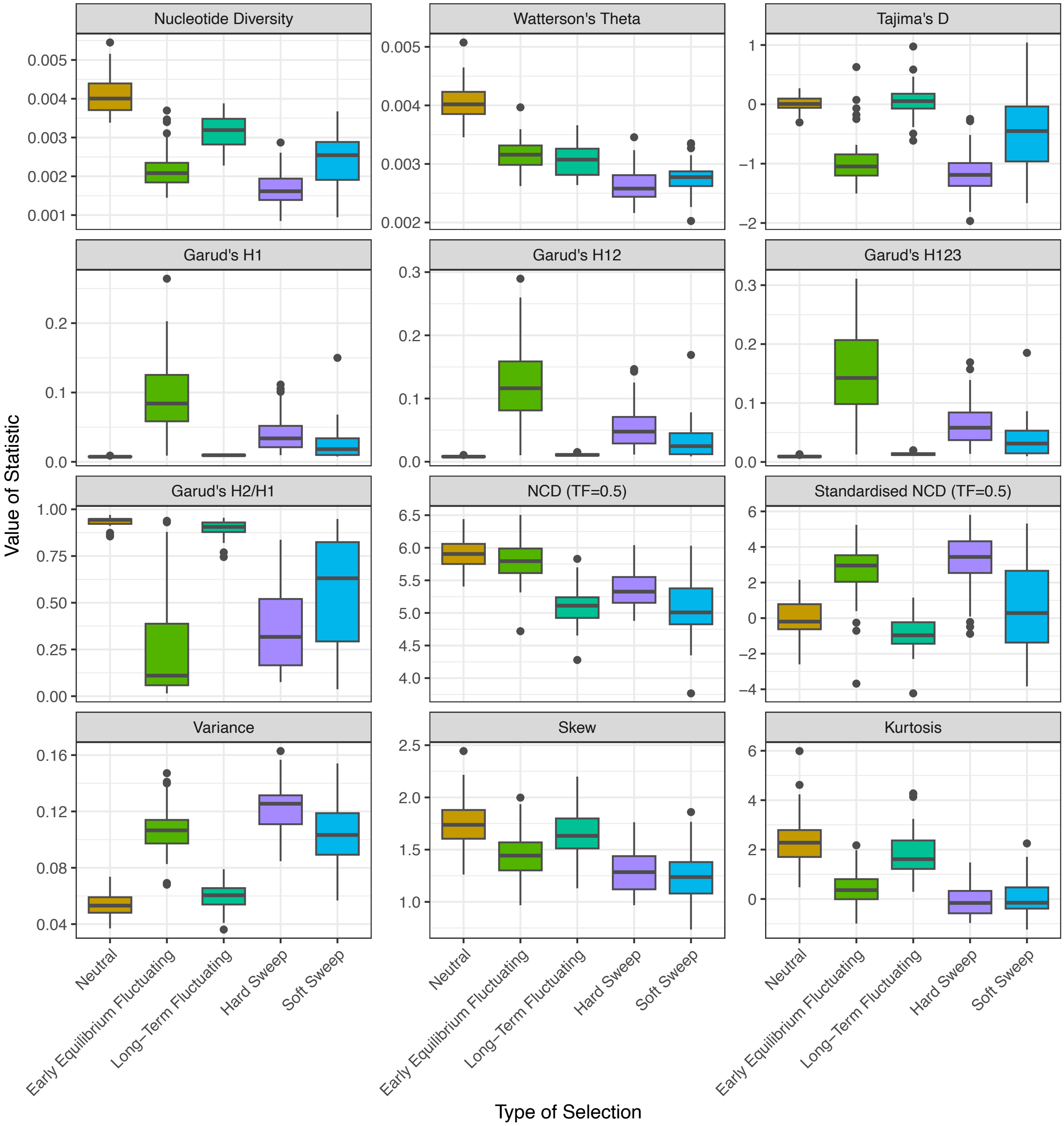

Figure S16. Population genetic statistics at the 10 kb window centered over the selected site for weak positive selection in the form of hard selective sweeps and soft selective sweeps (*s* = 0.1) and strong fluctuating selection (*s* = 1).

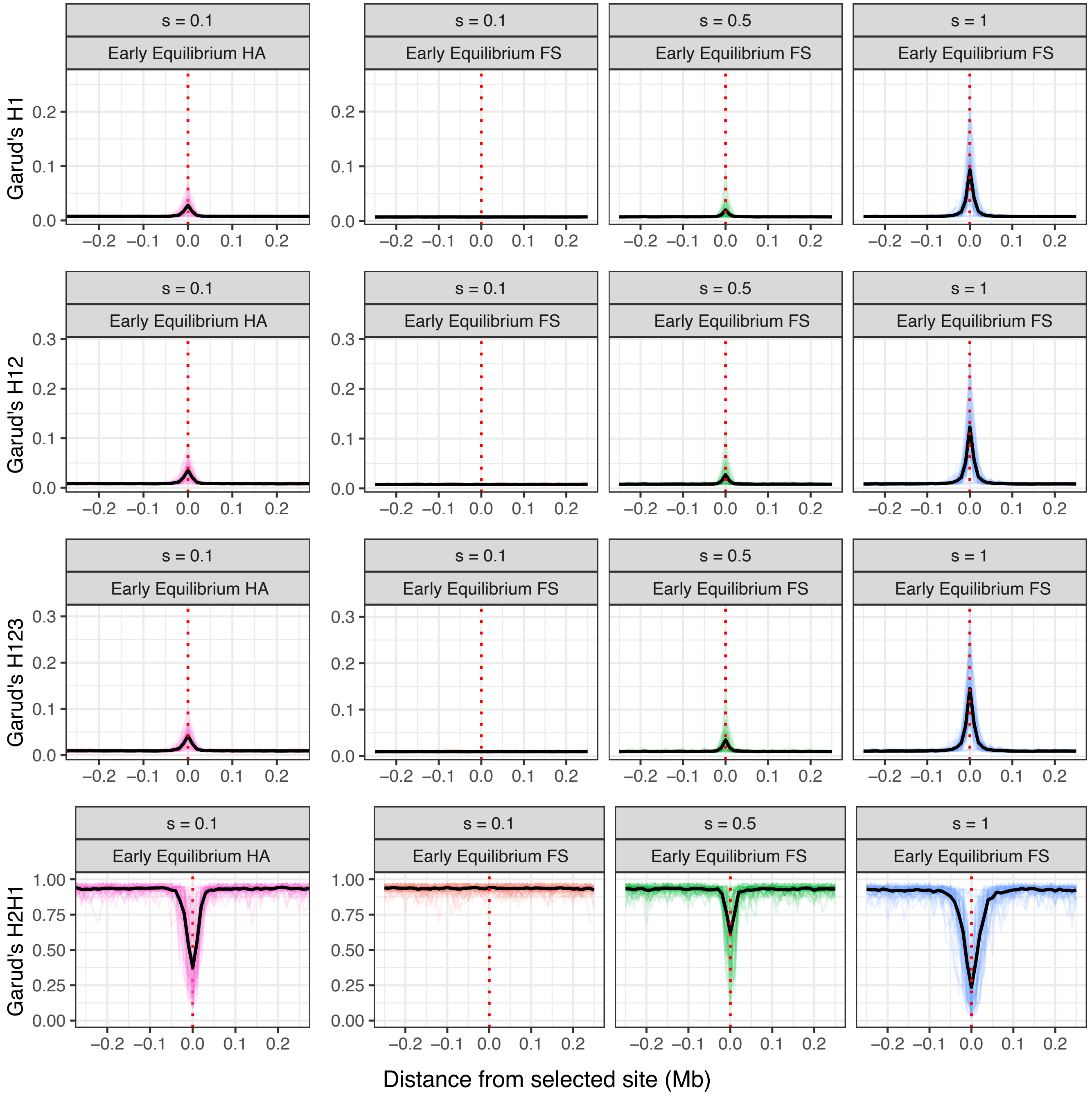

Figure S17. Comparisons of Garud's haplotype statistics between heterozygote advantage (HA; *s* = 0.1) and fluctuating selection of all selection coefficients (FS; *s* = 0.1, 0.5, 1). The average of 50 replicates is depicted by the black line, while the coloured lines visualize the replicates.

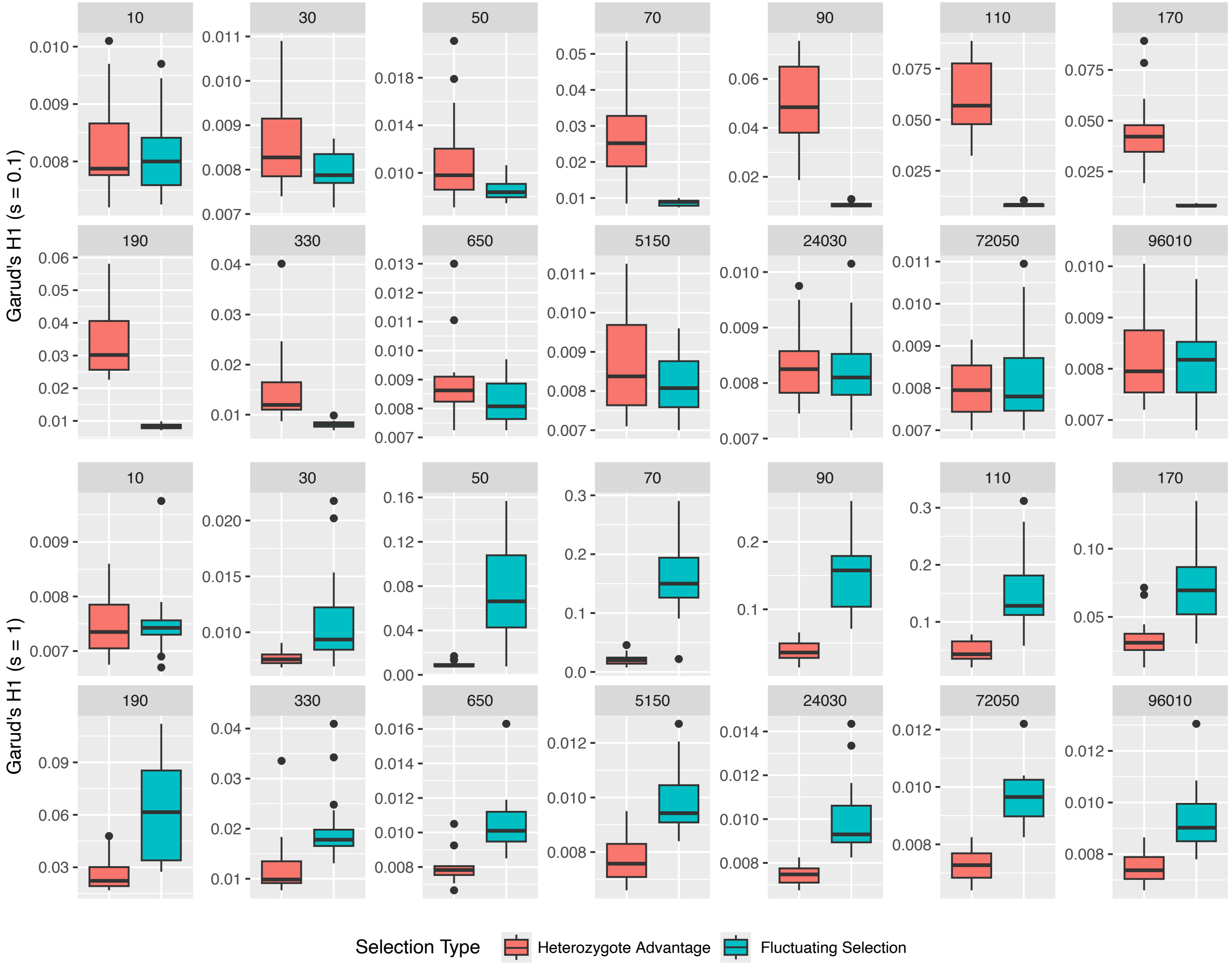

Figure S18. Distributions of fluctuating selection and heterozygote advantage over time for Garud's H1. Heterozygote advantage is shown in red and fluctuating selection in blue. The generation of the simulation is labelled above each facet. When $s = 0.1$, fluctuating selection and heterozygote advantage converge over time such that at long-term time points, signals are not significantly different. However, the respective signals do not converge within the simulated time frame when $s = 1$ for fluctuating selection.

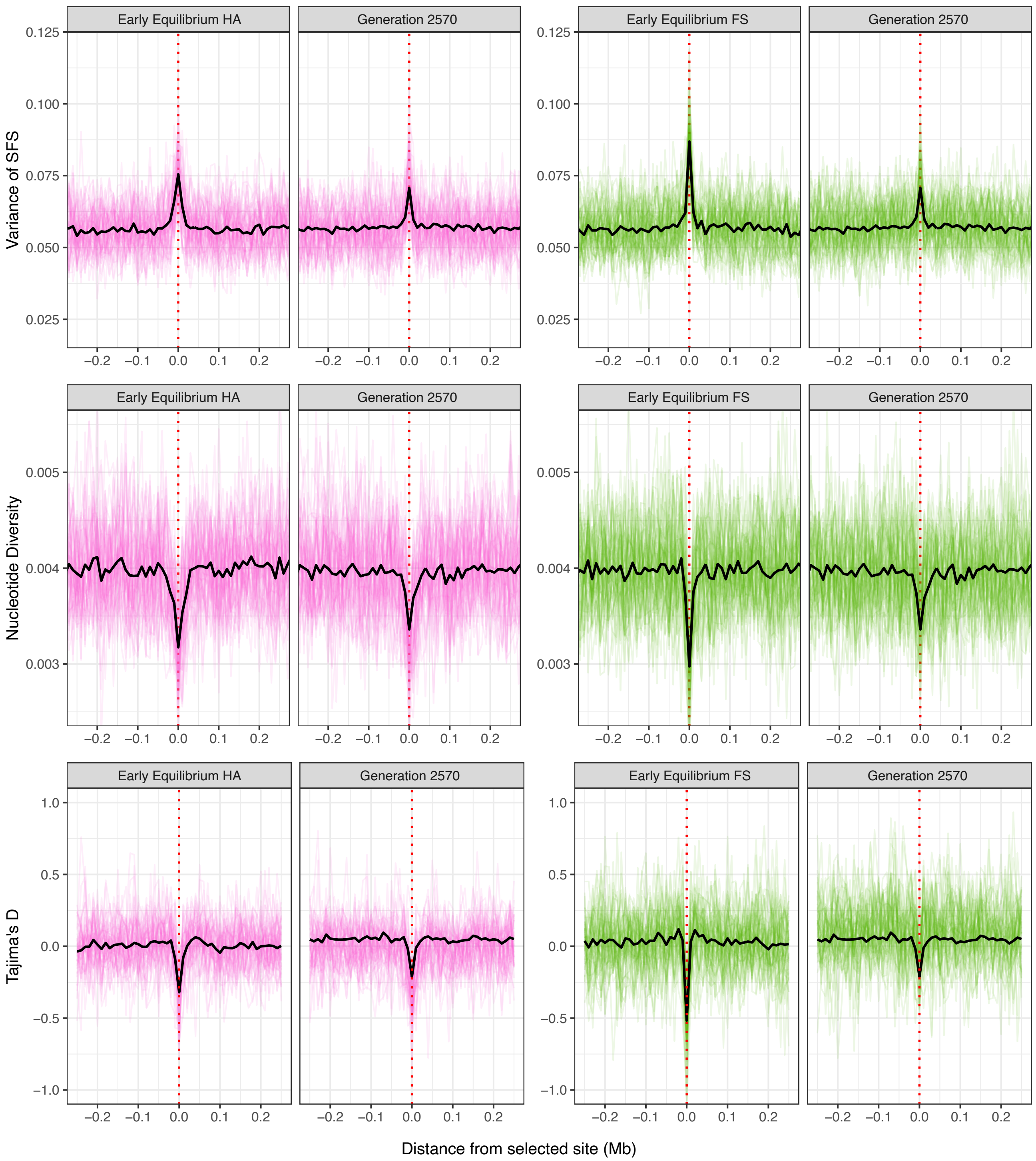

Figure S19. Heterozygote advantage (HA; *s* = 0.1) and fluctuating selection (FS; *s* = 0.5) when sampled at the conditional early equilibrium and the same time point (i.e. 2570 generations) for variance in the SFS, nucleotide diversity, and Tajima's D. Black lines show the average of all 50 replicates (shown in pink and green for heterozygote advantage and fluctuating selection, respectively.

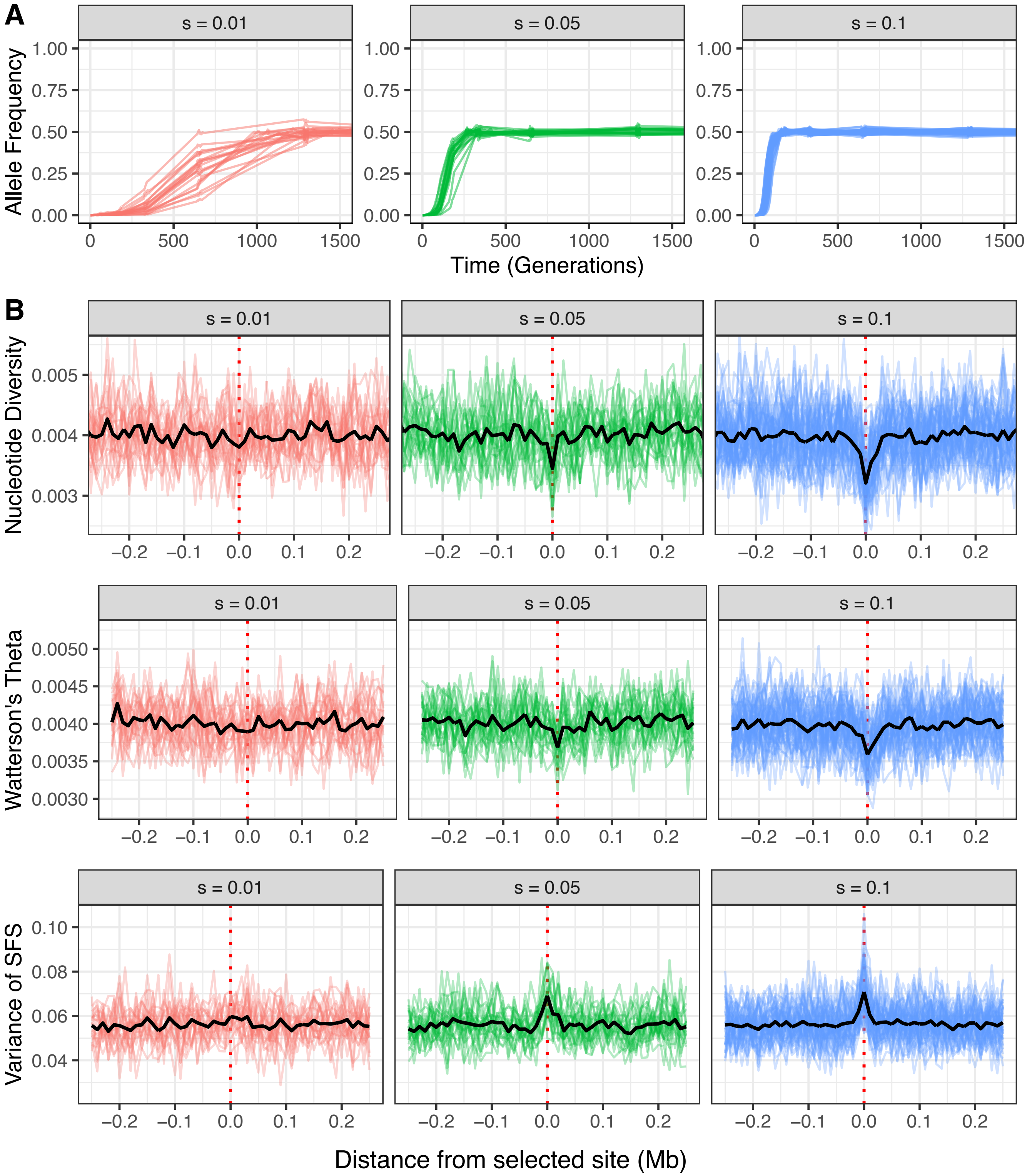

Figure S20. Heterozygote advantage at three selection coefficients (*s* = 0.01, 0.05, 0.1) with the selected allele frequency trajectories from *de novo* to stable equilibrium and associated variance in SFS, Watterson's theta and nucleotide diversity at equilibrium. Black lines show the averages of all 50 replicates, depicted by the coloured lines.

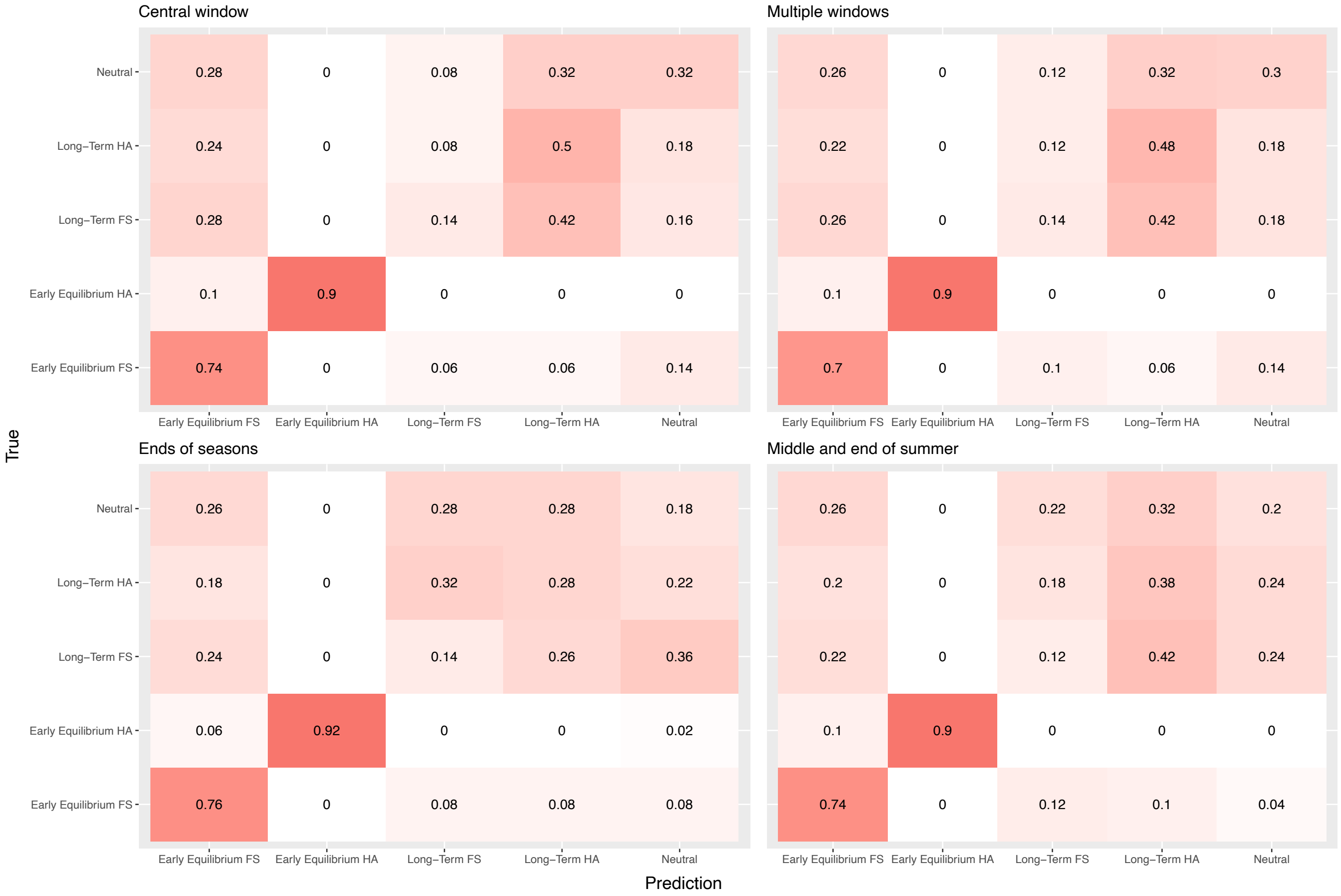

Figure S21. LDA model accuracy for fluctuating selection coefficient of 0.1.

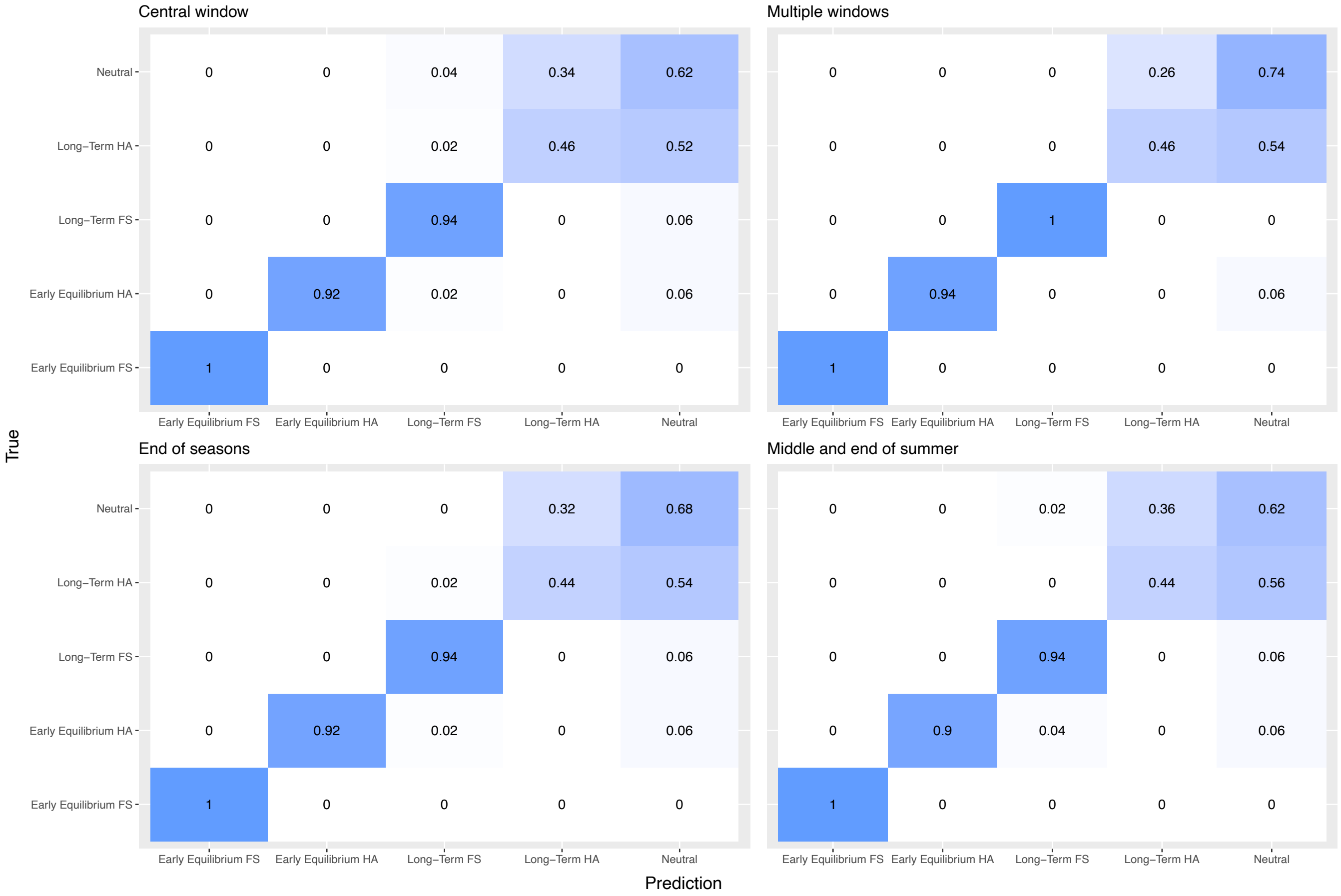

Figure S22. LDA model accuracy for fluctuating selection coefficient of 1.

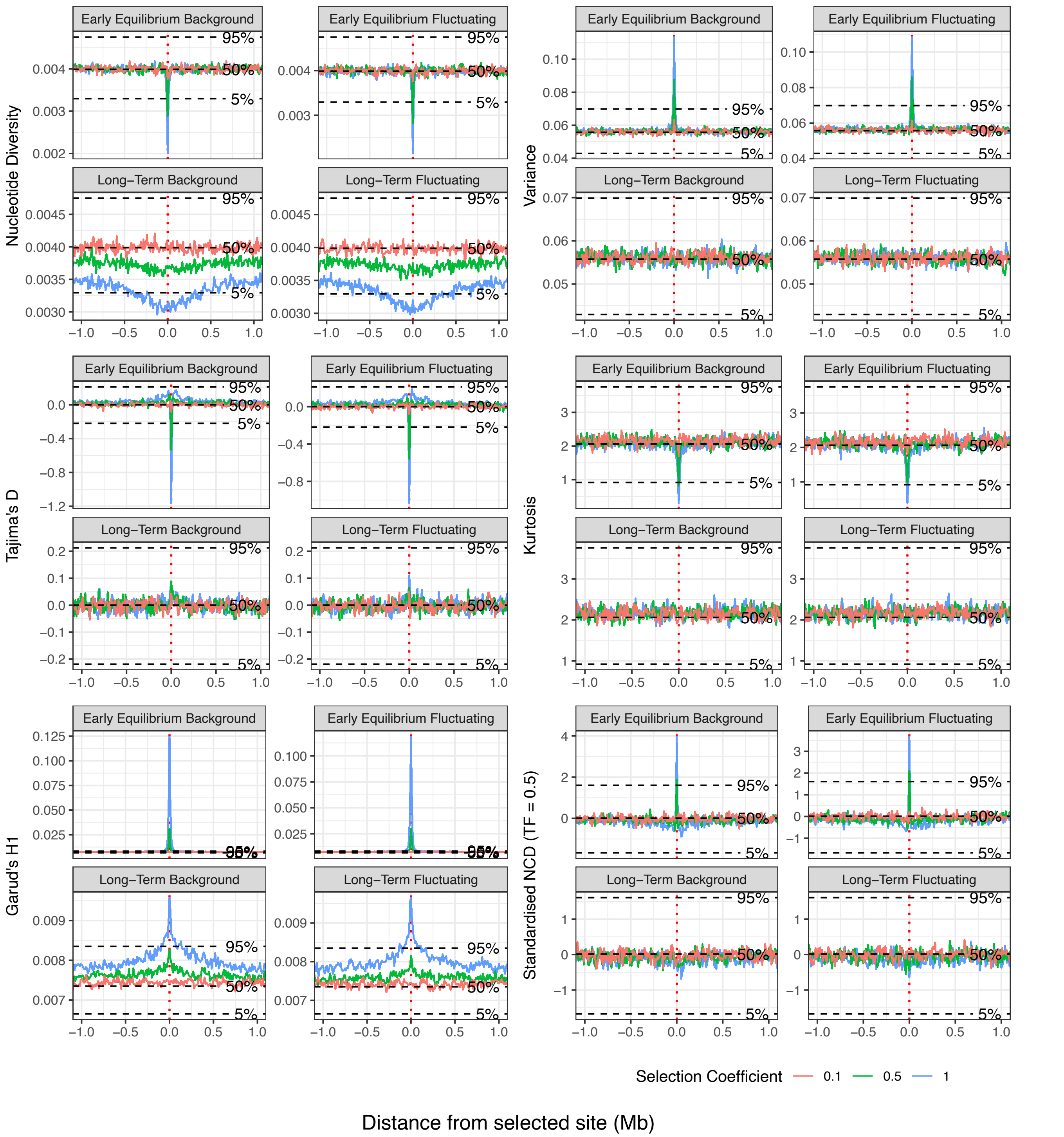

Figure S23. Distribution of population genetic statistics for fluctuating selection with and without background selection.

Panels show fluctuating selection at our Early Equilibrium and Long-Term time points with background selection (Early Equilibrium/Long-Term Background) and without background selection (Early Equilibrium/Long-Term Fluctuating). Statistics were calculated in 10kb windows across the simulated region and were averaged across 50 replicates for three selection coefficients: 1; 0.5; and 0.1, (see key). The vertical dashed red line signifies the position of the selected site. Horizontal dashed black lines illustrate the 5%, 50% and 95% quantiles of the 50 replicate values of each statistic for neutral simulations (denoted by label).

Appendix 1: Establishment probability

Simulations were restarted if the selected mutation was lost, with fluctuating selection having an increased number of restarts compared to heterozygote advantage and hard selective sweeps (Figure A1-1). Soft sweeps were not investigated due to their differential set-up. The selected allele was most often lost in the first few generations of the simulation for heterozygote advantage, fluctuating selection and hard selective sweeps. However, though the number of restarts dropped off after this initial peak, for fluctuating selection, the level remained elevated compared to other forms of selection for approximately the next 50 generations, particularly between generations 10 and 20 which was the binary winter season in which the summer-favored mutation is selected against (Figure A1-2).

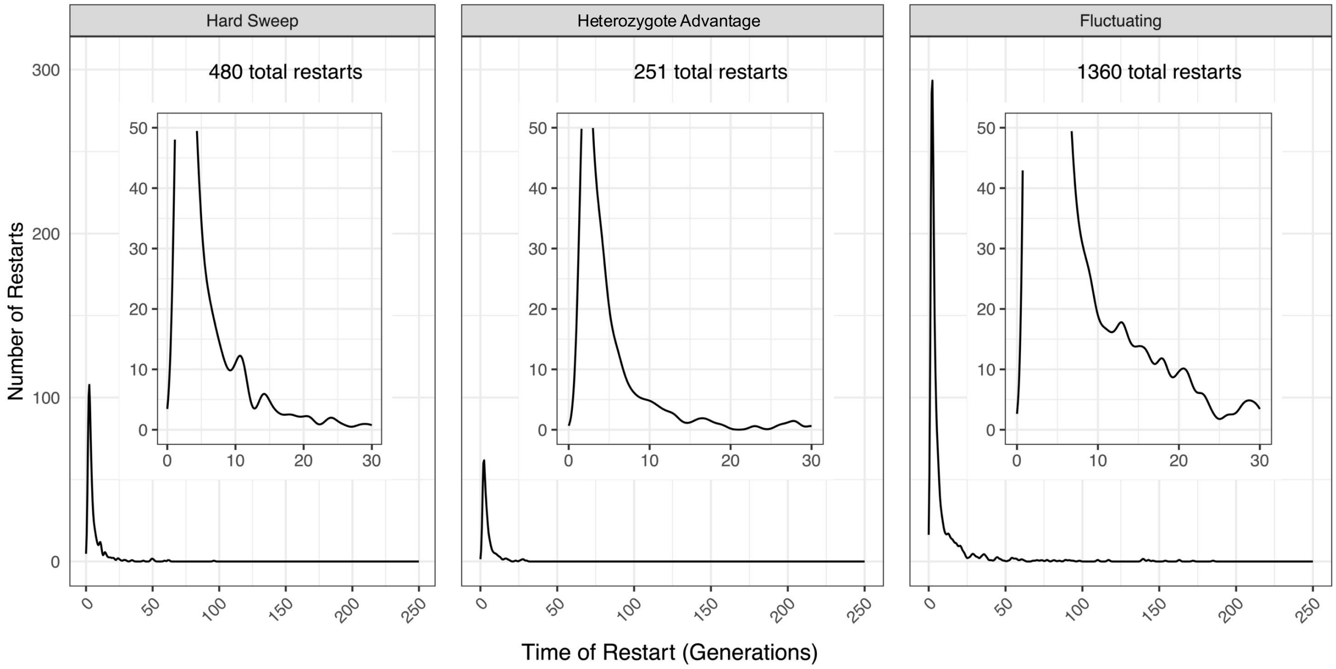

Figure A1-1. Establishment distribution of hard selective sweeps, heterozygote advantage and fluctuating selection with a selection coefficient of 0.1.

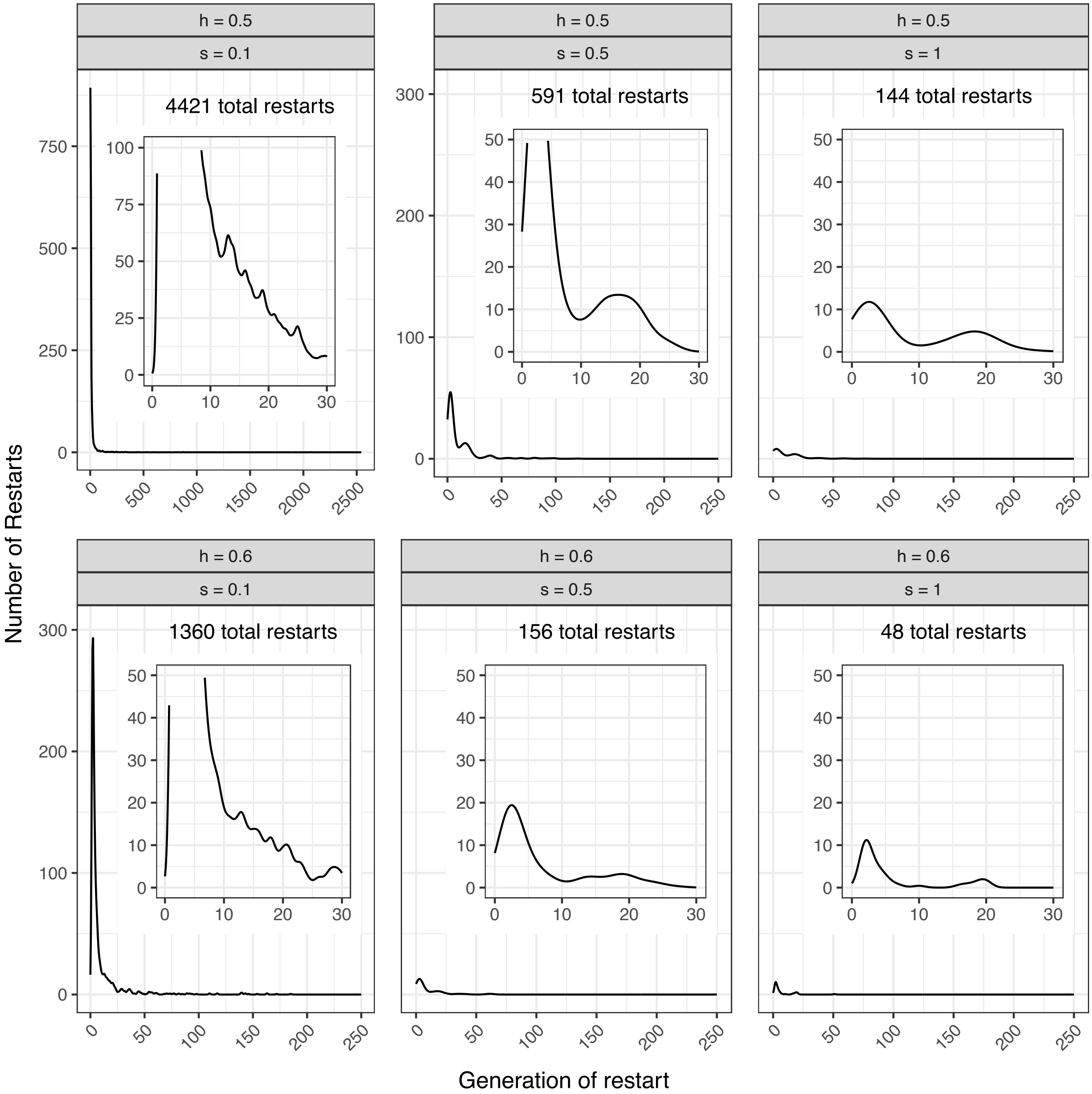

Figure A1-2. Establishment distribution of fluctuating selection for selection coefficients (*s*) 0.1, 0.5, 1 and dominance coefficients (*h*) 0.5, 0.6.

Appendix 2: Robustness of Scaling

To assess the robustness of our results to scaling and the broader relevance of our downscaled simulations for large population size species, we modeled a seasonally fluctuating locus in a population of 100,000 individuals (20 replicates, with parameters scaled to represent our N = 10,000, *s* = 1.0 simulations) and observed allele frequency fluctuations that matched that of the downscaled simulations included in our manuscript (see Figure A2-1). When examining the effects of fluctuating selection under these conditions on linked neutral genetic variation. Additionally, at the long-term timepoint we see the signature in the population is still transitioning from the early equilibrium signature to that found in the further downscaled simulations (Figure A2-2). Overall, we conclude that our downscaled simulations offer a conservative estimate of the effects of fluctuating selection on linked neutral genetic variation, as the observed signals are larger and more pronounced in simulations with a higher population size. Given that our LDA approach still accurately distinguishes fluctuating selection from other modes, we believe our downscaled simulations are adequate and provide a conservative estimate of the power to detect fluctuating selection in smaller populations.

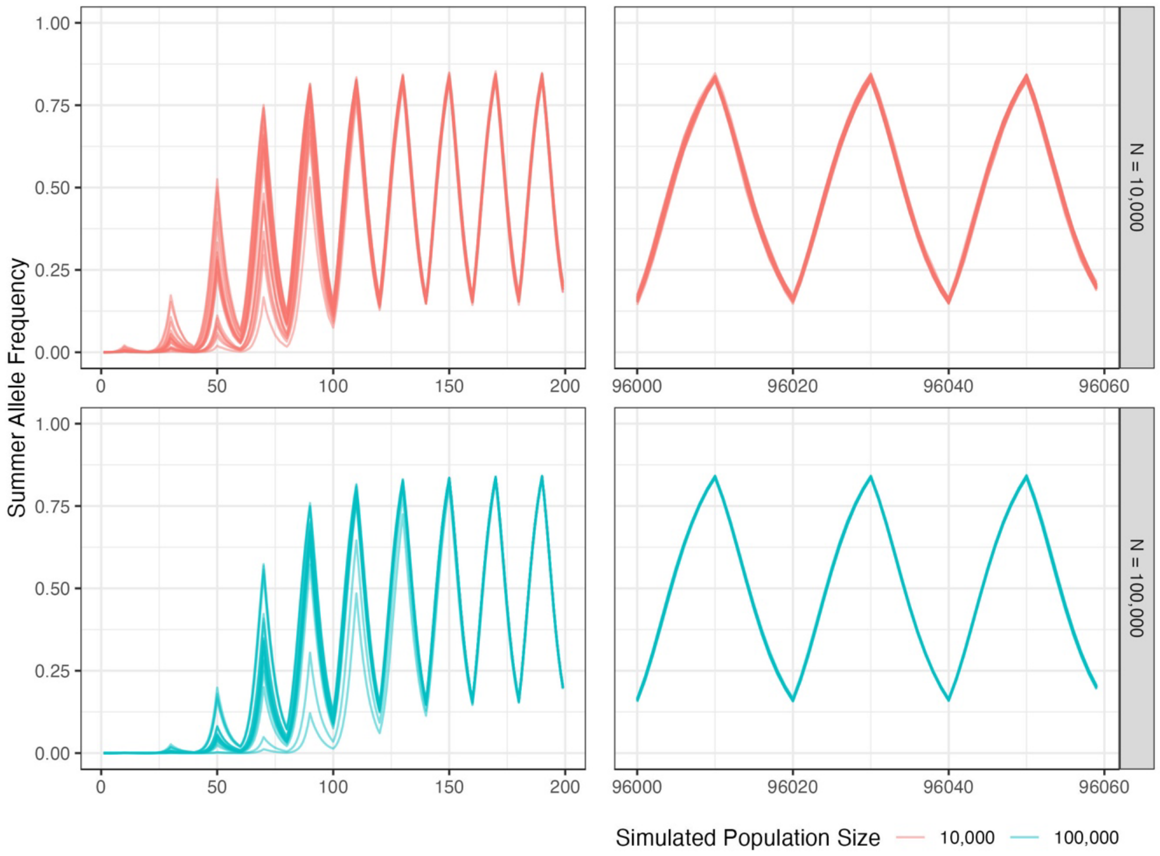

Figure A2-1. Allele frequency trajectories for downscaled simulations used in the manuscript (N = 10,000, shown in red) and appropriately upscaled simulations (N = 100,000, depicted in blue). The time for the fluctuations to stabilise at the early equilibrium time point and the magnitude of the fluctuations were matched between the two simulation parameters. 20 replicates were simulated for each set of parameters and are visualised here.

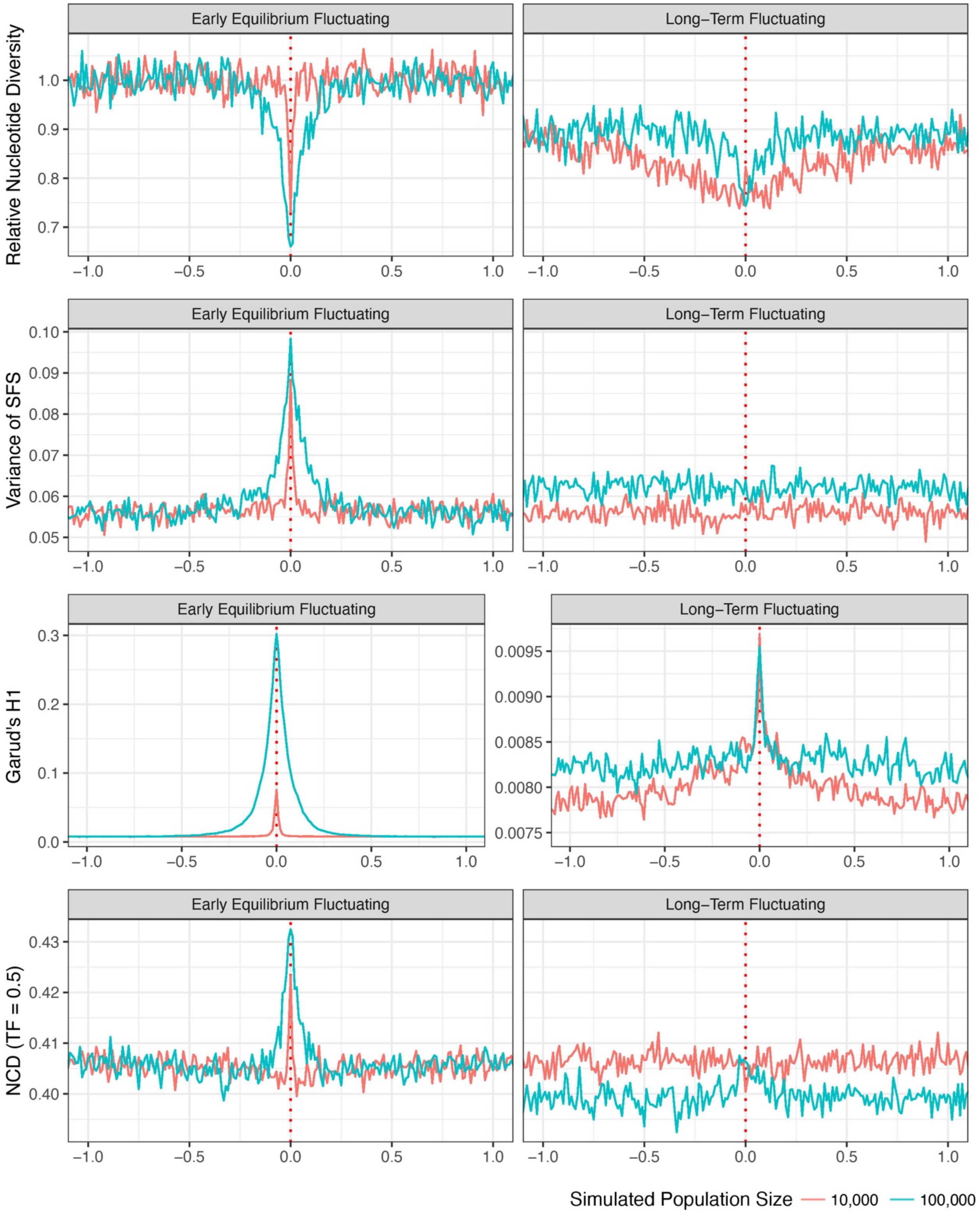

Figure A2-2. Effects of fluctuating selection on linked neutral genetic variation for downscaled simulations used in the manuscript (N = 10,000, shown in red) and appropriately upscaled simulations (N = 100,000, depicted in blue). 20 replicates were simulated for each set of parameters and average to formulate the depicted genomic signatures. We visualised the effect between the two parameter sets for relative neutral diversity, variance of the SFS, Garud's H1 and NCD (TF = 0.5).
